# Collaborative and privacy-enhancing workflows on a clinical data warehouse: an example developing natural language processing pipelines to detect medical conditions

**DOI:** 10.1093/jamia/ocae069

**Published:** 2024-04-04

**Authors:** Thomas Petit-Jean, Christel Gérardin, Emmanuelle Berthelot, Gilles Chatellier, Marie Frank, Xavier Tannier, Emmanuelle Kempf, Romain Bey

**Affiliations:** Innovation and Data Unit, IT Department, Assistance Publique-Hôpitaux de Paris, Paris, 75012, France; Innovation and Data Unit, IT Department, Assistance Publique-Hôpitaux de Paris, Paris, 75012, France; Institut Pierre-Louis d’Epidémiologie et de Santé Publique, INSERM, Sorbonne Université, Paris, 75012, France; Department of Cardiology, Hôpital Bicêtre, Assistance Publique-Hôpitaux de Paris, Le Kremlin Bicêtre, 94270, France; Innovation and Data Unit, IT Department, Assistance Publique-Hôpitaux de Paris, Paris, 75012, France; Department of Medical Informatics, Assistance Publique-Hôpitaux de Paris, Centre-Université de Paris (APHP-CUP), Université de Paris, Paris, 75015, France; Department of Medical Informatics, Hôpitaux Universitaires Paris-Saclay, Assistance Publique-Hôpitaux de Paris, Le Kremlin-Bicêtre, 94270, France; Laboratoire d'Informatique Médicale et d'Ingénierie des Connaissances pour la e-Santé (LIMICS), INSERM, Université Sorbonne Paris Nord, Sorbonne Université, Paris, 75005, France; Laboratoire d'Informatique Médicale et d'Ingénierie des Connaissances pour la e-Santé (LIMICS), INSERM, Université Sorbonne Paris Nord, Sorbonne Université, Paris, 75005, France; Department of Medical Oncology, Henri Mondor and Albert Chenevier Teaching Hospital, Assistance Publique-Hôpitaux de Paris, Créteil, 94000, France; Innovation and Data Unit, IT Department, Assistance Publique-Hôpitaux de Paris, Paris, 75012, France

**Keywords:** natural language processing, domain adaptation, Charlson score index, comorbidities, privacy

## Abstract

**Objective:**

To develop and validate a natural language processing (NLP) pipeline that detects 18 conditions in French clinical notes, including 16 comorbidities of the Charlson index, while exploring a collaborative and privacy-enhancing workflow.

**Materials and Methods:**

The detection pipeline relied both on rule-based and machine learning algorithms, respectively, for named entity recognition and entity qualification, respectively. We used a large language model pre-trained on millions of clinical notes along with annotated clinical notes in the context of 3 cohort studies related to oncology, cardiology, and rheumatology. The overall workflow was conceived to foster collaboration between studies while respecting the privacy constraints of the data warehouse. We estimated the added values of the advanced technologies and of the collaborative setting.

**Results:**

The pipeline reached macro-averaged F1-score positive predictive value, sensitivity, and specificity of 95.7 (95%CI 94.5-96.3), 95.4 (95%CI 94.0-96.3), 96.0 (95%CI 94.0-96.7), and 99.2 (95%CI 99.0-99.4), respectively. F1-scores were superior to those observed using alternative technologies or non-collaborative settings. The models were shared through a secured registry.

**Conclusions:**

We demonstrated that a community of investigators working on a common clinical data warehouse could efficiently and securely collaborate to develop, validate and use sensitive artificial intelligence models. In particular, we provided an efficient and robust NLP pipeline that detects conditions mentioned in clinical notes.

## Introduction

Recent scientific breakthroughs demonstrated the high potential of large health databases to generate new medical knowledge, but many challenges still remain to structure the emerging research and innovation ecosystems.^1^^,^^2^ These communities should exploit workflows which judiciously make use of new technologies to leverage data at scale while complying with the many constraints that limit data access to protect patients’ privacy. Clinical data warehouses (CDW) are new platforms that enforce these constraints by providing both secured technical environments and dedicated regulatory pathways. General CDW architecture could benefit from innovation, as no privacy-enhancing technology has provided a silver bullet solution yet.^3^ These needs for workflow and architecture innovation became even more obvious with the advent of new machine learning (ML) models such as large language models or foundation models. Indeed, training these models often requires accessing unminimized datasets, and some of the trained models have been proven privacy-sensitive as they leaked information related to patients’ records.^2^^,^^4^^,^^5^

These opportunities and challenges can be exemplified by algorithms that automatically detect conditions in clinical notes. Conditions—especially comorbidities—are routinely considered in clinical practice and epidemiological studies to identify disease combinations that may require a specific therapeutic approach or that are required for a proper population analysis.^6–8^ The Charlson comorbidity index (CCI) can for instance be computed from 16 conditions to predict the 1-year mortality of patients admitted in hospitals.^9^ Algorithms were developed to detect those conditions and compute the CCI using structured claim data.^10^^,^^11^ However, this data source was primarily designed for economic purposes and suffers from missingness issues. Nevertheless, it was shown that information could be efficiently obtained from clinical notes using natural language processing (NLP) algorithms instead, which rely more and more on ML techniques such as language models.^12–19^ Developing tools to this end remains challenging, and many difficulties are yet to be overcome for a wide community to benefit from them.^4^^,^^7^^,^^19–24^

First, no *one-size-fits-all* NLP technology exists, as optimal methods depend on the specificity of each setup, notably on tasks, available data, context, regulatory, and computational limitations, etc. Although ML approaches have been proven efficient for some tasks, an architecture based on the transposition of expert clinical knowledge in explicit rules still often reaches similar or better performances.^7^^,^^25^ Hybrid approaches combining rule-based and ML techniques are a promising field of research in order to benefit from the strengths of both techniques and optimally leverage the expertise of clinicians and computer scientists.^26^ Additionally, sharing hybrid pipelines is facilitated as privacy sensitive and non-sensitive modules can be kept distinct. Second, NLP algorithms developed in a study are often difficult to apply to new contexts. Beyond the obvious challenge of reusing algorithms developed for other languages, each specialty domain may moreover feature specific syntaxes and vocabularies that impact the algorithms’ robustness.^21^^,^^27^ Consequently, it appears sound to develop solutions jointly considering different datasets to enhance generalizability. Third, it would be relevant to associate various medical expertises to the development, but investigator data access is often restricted to the disease-specific cohorts corresponding to their areas of research. Therefore, new privacy-enhancing workflows should be implemented to enable large communities to efficiently collaborate.^5^^,^^7^^,^^16^^,^^28^^,^^29^

To address these entangled technical and organizational issues, we developed, validated, and shared a hybrid NLP pipeline that detects in clinical notes written in French the 16 conditions of the CCI, along with the tobacco and alcohol consumption statuses. Leveraging the hybrid architecture of this pipeline, we adopted a *development-training-validation-serving* workflow that enabled the association of investigators working on 3 disease-specific cohorts while limiting privacy exposure. We estimated the performance gains provided by the advanced NLP technologies and by the collaborative setting, and we evaluated the reduction of privacy exposure provided by our workflow compared to simpler alternatives.

## Materials and methods

Our study involved clinicians and data from 3 retrospective observational studies reviewed and approved by the Institutional Review Board of our institution (*IRB*00011591, decisions *CSE*18-32, *CSE*20-55, and *CSE*20-93) and re-used a large language model trained on 21 million pseudonymized clinical notes in the context of a previous study (decision *CSE*19-20).^20^ French regulation does not require the patient’s written consent for this kind of research, but the patients were informed of this research, and those who objected to the secondary use of their data were excluded from the study.^30^ Data was pseudonymized by replacing names and places of residence by aliases. We followed the REporting of studies Conducted using Observational Routinely-collected health Data reporting guideline (see checklist in Supplementary Materials).^31^

### Rheumatology, oncology, and cardiology cohorts

Each study’s dataset is constituted of an extraction of the overall database containing routine data of patients cared for in the *Greater Paris University Hospitals* (Assistance Publique-Hôpitaux de Paris, AP-HP, see Supplementary Appendix A for details on cohort selection). To limit privacy exposure, each investigator accessed only her study’s data (see Figure 2).

We considered demographic data (age at admission, gender), diagnostic claim codes, discharge summaries (for inpatient stays), and consultation reports (for outpatient stays). To avoid data leakage, we followed *Bey et al* methodology and divided each cohort into a training cohort and a validation cohort (see Figure S1).^32^

### Data sources

AP-HP comprises 38 university hospitals spread across the Greater Paris area (more than 22 000 beds, 1.5 million hospitalizations each year) which use a common electronic health record (EHR) software (ORBIS, Dedalus Healthcare). Data collected in the EHR and in the claim database (French PMSI, *Programme de Médicalisation des Systèmes d’Information*, coded following the ICD-10, the *International Classification of Diseases* 10*th revision*) are integrated in the AP-HP CDW on a daily and monthly basis, respectively. The research database follows the *Informatics for Integrating Biology & the Bedside* standard.^33^ Data were extracted for this study on April 2, 2021, July 13, 2021, and October 10, 2021 for the rheumatology, cardiology, and oncology cohorts, respectively.

### Conditions definitions

We considered the 16 conditions of the CCI. Separately, we also extracted the tobacco and alcohol consumption statuses. These additions followed both a medical rationale (ie, the variables were deemed clinically relevant although they would not fit in the original CCI) and an opportunistic rationale (ie, to make the most of the annotation process). For consistency, they were not considered when computing the CCI. The definitions of these conditions were refined by clinicians (C.G., E.K., E.B.) to optimally balance their relevance for epidemiological research (eg, avoiding asymptomatic conditions) and the reliability of their detection in clinical notes (eg, avoiding excessive interpretation of textual mentions). Each condition was defined as a free text, a list of ICD-10 codes inspired by *Sundararajan et al* and a machine-readable dictionary of synonyms expressed as regular expressions with composition rules (see Figure S2).^11^ When present, 5 conditions displayed 2 possible statuses, namely diabetes (With end-organ damage/Uncomplicated), liver disease (Moderate to severe/Mild), solid tumor (Metastatic/Localized), and tobacco or alcohol consumption (Present/Stopped). An annotation guideline details the annotation methodology (see Supplementary Appendix E).

### Pipelines architecture

**Figure 1.**
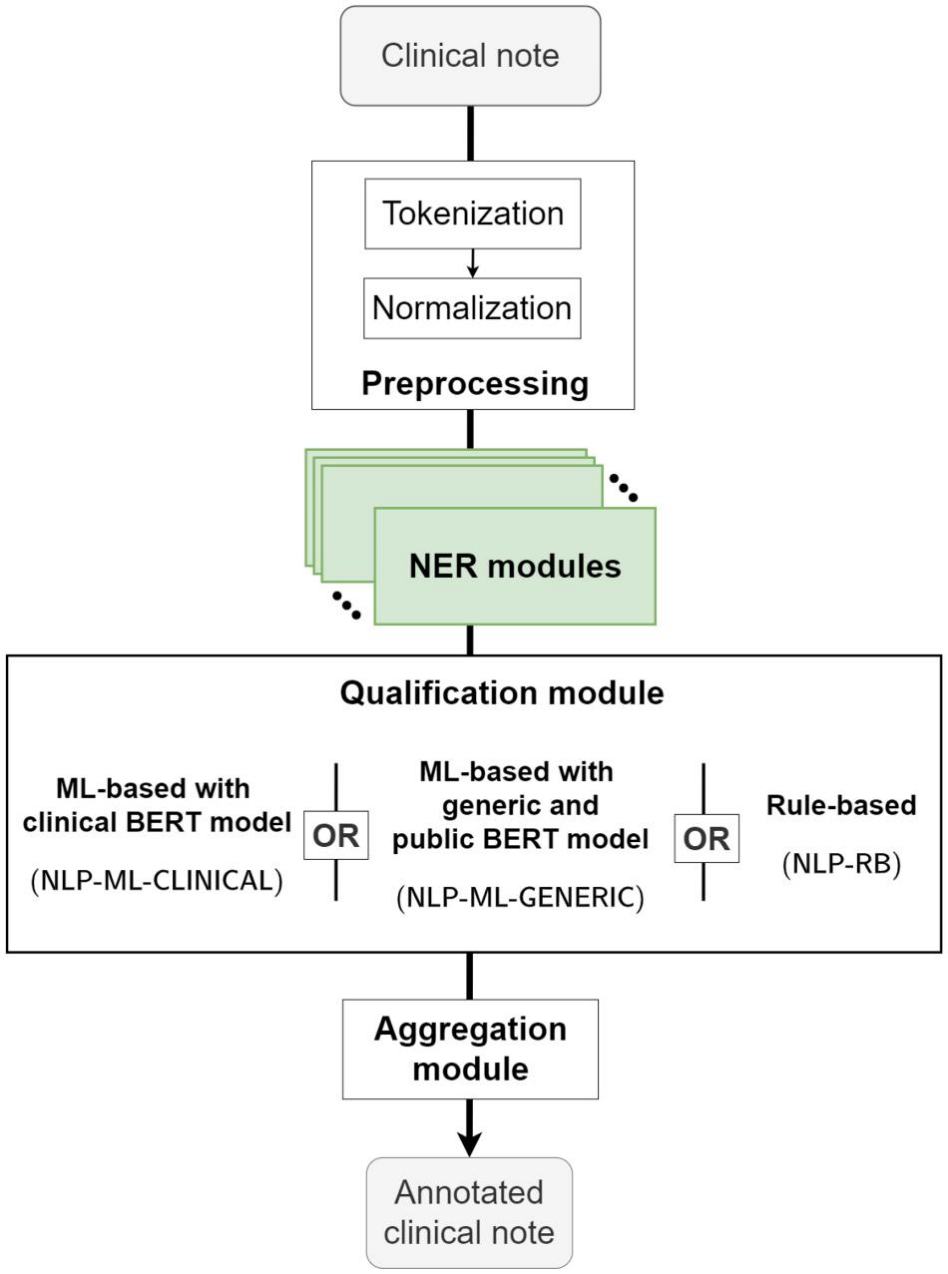
Structure of the 3 implemented natural language processing pipelines (NLP-ML-CLINICAL, NLP-ML-GENERIC, and NLP-RB), each featuring a specific Qualification module. In green are the rule-based, iteratively-improved Named Entity Recognition modules.

We are referring to the main NLP pipeline that we developed as NLP-ML-CLINICAL. It consisted of 18 Named Entity Recognition (NER) modules that extract mentions of each condition in the clinical note, followed by common qualification and aggregation modules (see Figure 1). An upstream preprocessing module tokenizes and normalizes text from the note by switching to lowercase, removing diacritics, standardizing quotes, and detecting irrelevant sections (see Supplementary Appendix B for details).

Each NER module relied on a curated dictionary of regular expressions for each condition. Additional rules were implemented to deduce, if applicable, the condition’s severity from the entity’s context (ie, a snippet of words around the extraction, the size of which varies between modules) and to discard false positives caused by identified mechanisms (eg, an incorrect acronym). Modules were developed within the open source EDS-NLP scientific library and were made available in its 0.8.1 release.^34^

The Qualification module was a machine-learning module responsible for discarding irrelevant entities. We focused on detecting negation and speculation—an unavoidable step when performing information retrieval in clinical texts—along with family history, necessary here since our work aims at extracting conditions.^35^ To this end, entities were labeled as irrelevant during annotation if they were either negated (*the patient has no diabetes*), hypothetical (*the patient may have diabetes*), or not related to the patient (*the patient’s father had diabetes*). Generic false positives (eg, a condition mentioned in a care site name or in a laboratory measure denomination) were also labeled as irrelevant. The module was based on EDS-CamemBERT, a clinical language model that was trained on 21 millions pseudonymized clinical notes from the AP-HP’s CDW.^20^ This architecture took a snippet of text containing an entity as input and sequentially (1) generated embeddings for each token of the snippet, (2) mean pooled the embeddings of the entity’s tokens, and (3) classified the pooled embedding using a fully-connected layer, thus predicting if the entity should be discarded or not (see Figure S3 for the detailed architecture).

The Aggregation module predicted the conditions at the stay level out of the list of detected and qualified entities in its associated clinical note. For binary predictions, the module predicted the presence of the condition if at least 1 entity was detected without being discarded. For severity predictions, the module predicted the status of the most severe detected entity.

For comparison, we also developed a set of alternative pipelines. The NLP-ML-GENERIC pipeline replaced the EDS-CamemBERT model from NLP-ML-CLINICAL with CamemBERT, a publicly available and generic language model.^36^ The NLP-RB pipeline explored a rule-based approach for the qualification module, implementing 3 separate algorithms to detect negation, hypothesis, and familial relations. Finally, for inpatient stays only, we developed the CLAIM pipeline which extracted conditions from claim codes (See Supplementary Appendix B).

### Pipelines development and training

**Figure 2.**
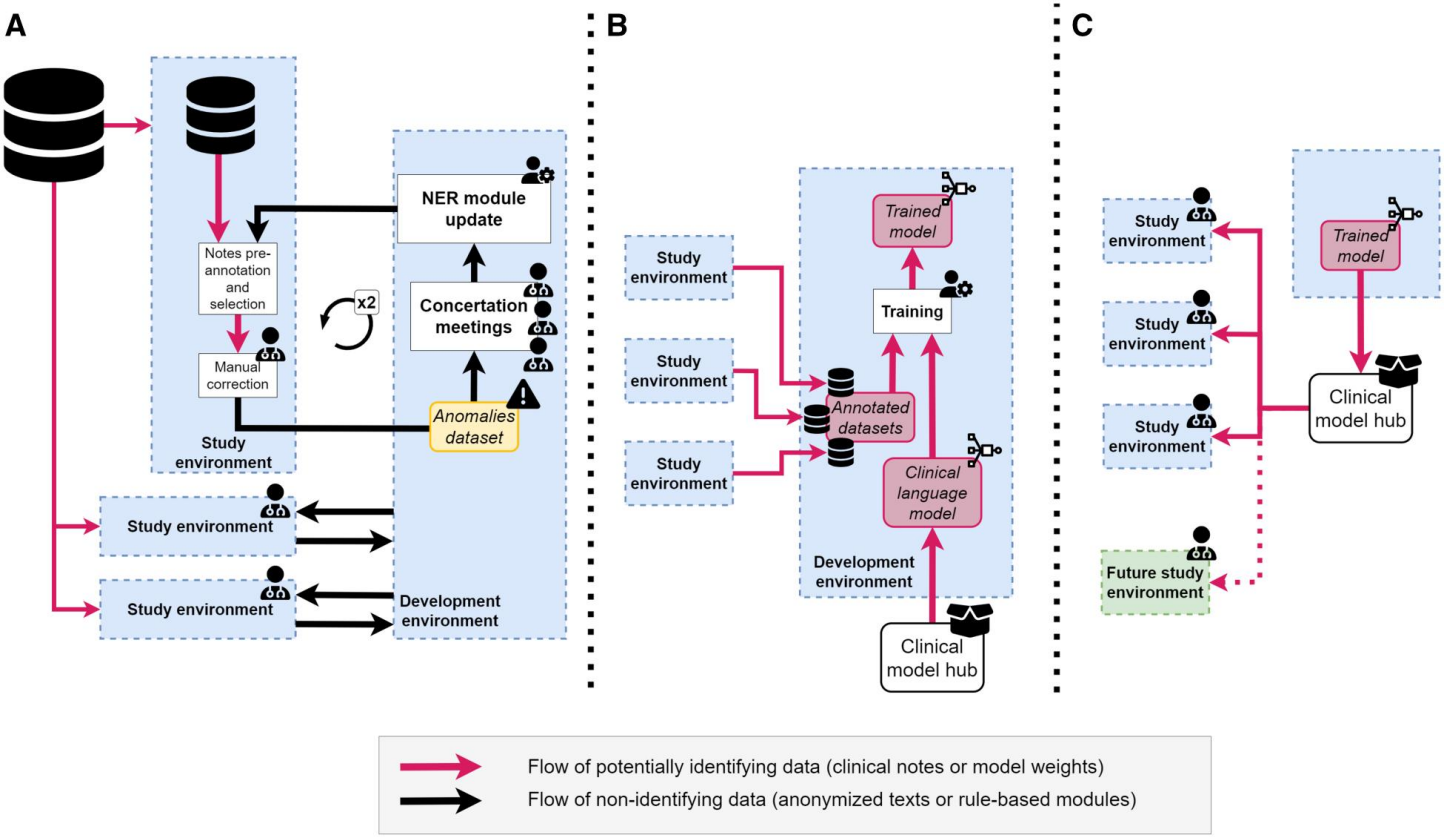
Development, training, and serving workflows. (A) Iterative development of the rule-based named entity recognition (NER) module of the natural language processing (NLP) pipeline: the 2 annotation sessions take place in each 1 of the 3 studies’ secure environments accessing the full electronic health records of each cohort’s patients, while development occurs in a separate dedicated environment accessing only non-identifying anomalies. (B) Training of the machine learning (ML) based qualification module: the module is initialized by a clinical privacy-sensitive model downloaded from a secured clinical model hub. It is then trained on snippets gathered during (A). (C) Validation and serving of the module: Once trained, the qualification module is pushed to the clinical model hub, from which it can be served to study environments.

The NER modules and annotations guidelines were jointly initialized by the 3 expert clinicians (C.G., E.K., E.B.) and the data scientist (T.P.-J.). Two annotation sessions ranging from about 1-2 hours were then organized in the 3 study environments to improve the pipeline’s performance. At the beginning of each session we used NLP-RB, which did not require any training and is not privacy sensitive, to pre-annotate clinical notes. A subset of clinical notes were randomly selected in the training cohorts adopting an upsampling strategy that optimized their relevance (see Supplementary Appendix C). Each clinician read the selected notes relative to her disease-specific cohort and confirmed or invalidated the entity-level predictions while adding entities that would have been falsely omitted by the modules. At the end of each session, concertation meetings were held to discuss clinical and methodological issues, and to improve the conditions’ definitions and dictionaries. Improvements could include, but were not limited to, adding diseases’ synonyms, performing acronym disambiguation or refining the medical rationale. We emphasize that this collaborative improvement of the rule-based NER was fully privacy preserving, as only limited, non-identifying anomalies were shared during these meetings (see Figure 2A).

Once the 2 annotation sessions were completed, the annotated entities and their contexts were reviewed to fit the consolidated definitions. The 3 annotated datasets (one per study environment) were sent to a common development environment, while the pre-trained clinical language model was downloaded in the development environment from a model hub (see Table S10 for a description of this training dataset). The environment was then accessed by the data scientist to train the ML qualification module (see Figure 2B). The clinical language model was imported from a private and secure clinical model hub after a prior validation by the IRB. Only 1 person had to access the development environment that moreover only contained annotated entities, their context, and clinical models. This access and data limitation enhances the privacy of our workflow.

### Pipelines validation

The source code and trained parameters of the NLP pipelines were frozen at the beginning of the validation session, and the qualification module was made available through the clinical model hub (see Figure 2C). The validation sub-cohorts were divided into inpatient and outpatient stays. The pipeline was applied to pre-annotate either the last-edited discharge summaries of inpatient stays or the consultation reports of outpatient stays. Each clinician reviewed notes drawn randomly in her disease-specific sub-cohort. After the validation session, 2 conditions (hemiplegia and AIDS) were found in fewer than 10 clinical notes overall. For those 2 conditions, we performed when possible an additional validation step by upsampling up to 30 clinical notes on which each condition was extracted by the pipeline. Due to this biased note selection, we only reported the positive predictive value (PPV) for these conditions. They were excluded from the aggregated metrics.

For every other condition, we reported the F1-score, the PPV, the sensitivity, and the specificity. In the case of conditions with 2 possible statuses, we considered the extraction of each status as a separate binary NER module and also reported the performance of the binary NER module consisting of detecting the presence of the condition, whatever its status. Entity-level (ie, the pipeline minus the aggregation module) and stay-level (ie, the full pipeline) predictions were both confirmed or invalidated by a chart review of the analyzed notes, thus leading to both an entity-level and a stay-level gold standard dataset. Regarding entity-level performances, we considered a prediction as correct if it overlapped with a gold standard entity.

We moreover assessed the usability of these pipelines to compute the CCI score. Indeed, although its computation is not the objective of the study, it may be carried out using the delivered pipelines. We compared the CCI computed using NLP-ML-CLINICAL with the same score computed using either the CLAIM pipelines or with its value directly mentioned in a subset of ICU notes by the clinicians at the point of care (see Supplementary Appendix D).

### Comparison with alternative technologies and collaboration settings

First, we compared the performance of our pipeline with alternative technologies and settings. For each condition we compared the performance of NLP-ML-CLINICAL with the 3 alternative pipelines NLP-ML-GENERIC, NLP-RB, and CLAIM. We also evaluated the added value of the collaborative setting on the qualification module by training and validating NLP-ML-CLINICAL separately in the 3 study environments. These performances were compared on the inpatient and outpatient stays of the validation sub-cohorts. Finally, to assess the generalizability of this pipeline, we evaluated its performance when trained and validated on distinct disease-specific cohorts.

Second, we evaluated the privacy enhancement provided by our workflow. We compared it with similar *development-training-validation-serving* workflows that would take place in alternative, more classical settings: (1) the non-collaborative setting discussed above where clinicians and data scientists work in isolated study environments, each 1 of them accessing only 1 cohort’s EHRs, and (2) a collaborative setting that would not leverage different environments to limit privacy exposure, ie, in this setting all the clinicians and data scientists access a single environment containing all the EHRs of the 3 cohorts (see Figure S4). We considered that privacy exposure increased with the number of persons accessing a patient’s EHR and its derivative data, ie, snippets of texts and trained models. However, accessing derivative data was less privacy-exposing, as it is usually impossible to re-identify a patient using only a snippet of text, and accessing only a model trained on these snippets makes a re-identification attack even less probable. The Supplement provides a synthetic view of privacy exposure in our workflow and in the 2 alternative settings (see Table S4) along with a more detailed description of the re-identification attacks that we intended to prevent.

## Results

Table 1 shows the number of stays and demographic characteristics of the validation sub-cohorts. Overall, 134 (89%) discharge summaries had at least 1 valid condition mentioned, on which an average of 10.1 entities were present. Similarly, 112 (75%) consultation reports had at least 1 valid condition mentioned, with 3.7 entities on average. When also considering discarded entities (eg, negated or hypothetical), those numbers of discharge summaries and consultation reports increased to 143 and 119, respectively, and the corresponding average number of entities increased to 13.2 and 4.4, respectively (see Table S5). Regarding discharge summaries, routinely reported conditions such as alcohol or tobacco consumption featured the highest increase, from 14 and 35 to 45 and 68, respectively.

**Table 1.** Composition of the validation dataset sampled in the 3 disease-specific cohorts.

Stay type	Cardiology cohort		Oncology cohort		Rheumatology cohort		Overall cohort	
	Inpatient	Outpatient	Inpatient	Outpatient	Inpatient	Outpatient	Inpatient	Outpatient
Age at admission	84.6 (7.3)	80.9 (6.9)	61.4 (18.7)	61.9 (15.2)	57.5 (19.4)	53.4 (19.2)	67.8 (20.0)	65.4 (18.6)
Gender distribution (M-F)	48-52	48-52	60-40	46-54	50-50	36-64	52-47	43-56
Total annotated records	50	50	50	50	50	50	50	50
Myocardial infarction	12 (3.3)	9 (2.1)	8 (3.0)	2 (1.0)	8 (3.1)	2 (2.0)	28 (3.2)	13 (1.9)
Congestive heart failure	34 (6.0)	21 (2.7)	11 (4.5)	1 (4.0)	13 (4.9)	2 (1.0)	58 (5.5)	24 (2.6)
Peripheral vascular disease	37 (1.8)	17 (1.9)	25 (2.2)	7 (1.3)	28 (2.5)	10 (2.2)	90 (2.1)	34 (1.9)
Cerebrovascular disease	9 (1.7)	4 (1.8)	3 (1.7)	2 (1.5)	12 (4.9)	3 (1.3)	24 (3.3)	9 (1.6)
Dementia	13 (1.6)	2 (2.5)	3 (1.3)	0	9 (2.8)	3 (1.3)	25 (2.0)	5 (1.8)
Chronic pulmonary disease	18 (3.9)	14 (1.6)	5 (3.8)	4 (1.5)	10 (4.2)	7 (1.7)	33 (4.0)	25 (1.6)
Rheumatologic disease	1 (1.0)	3 (1.3)	3 (2.7)	1 (2.0)	13 (4.5)	12 (1.7)	17 (4.0)	16 (1.6)
Peptic ulcer disease	6 (1.3)	0	3 (1.7)	1 (1.0)	2 (1.0)	0	11 (1.4)	1 (1.0)
Liver disease	0	1 (3.0)	2 (4.5)	1 (2.0)	7 (5.0)	2 (2.5)	9 (4.9)	4 (2.5)
Diabetes	15 (1.9)	13 (1.3)	12 (1.9)	4 (1.8)	10 (3.8)	5 (1.6)	37 (2.4)	22 (1.5)
Hemiplegia^a^	1 (2.0)	1 (2.0)	1 + *10* (4.0)	0 + *13* (-)	2 + *11* (1.0)	0 + *12* (-)	4 + *21* (2.0)	1 + *25* (2.0)
Renal disease	9 (1.9)	4 (2.0)	5 (1.2)	1 (3.0)	1 (3.0)	0	15 (1.7)	5 (2.2)
Solid tumor	7 (2.7)	6 (2.0)	21 (3.1)	21 (2.0)	7 (1.6)	6 (1.5)	35 (2.7)	33 (1.9)
Leukemia	1 (5.0)	1 (4.0)	3 (1.3)	2 (2.5)	4 (3.2)	2 (2.0)	8 (2.8)	5 (2.6)
Lymphoma	5 (2.8)	5 (1.6)	4 (4.8)	3 (2.7)	3 (2.7)	1 (1.0)	12 (3.4)	9 (1.9)
AIDS^a^	0	0	1 + *7* (2.0)	0 + *8* (-)	0 + *9* (-)	0 + *9* (-)	1 + *16* (2.0)	0 + *17* (-)
Alcohol consumption	3 (1.3)	1 (1.0)	5 (2.4)	1 (2.0)	6 (3.3)	1 (1.0)	14 (2.6)	3 (1.3)
Tobacco consumption	12 (1.7)	6 (1.5)	9 (1.8)	8 (1.1)	14 (1.9)	4 (1.0)	35 (1.8)	18 (1.2)
Total	48 (11.1)	40 (5.2)	42 (7.7)	37 (2.8)	44 (11.4)	35 (2.9)	134 (10.1)	112 (3.7)

For each condition, the number of clinical notes with at least 1 validated entity is shown along with, for those notes, the mean number of validated entities per note (in brackets). For instance, in the cardiology cohort, 12 discharge summaries mention a myocardial infarction, and on average 3.3 validated entities are present within each one of them.

a For these conditions, the number of upsampled notes is also shown *in italics*.

Table 2 shows stay-level performances of the NLP-ML-CLINICAL pipeline. Outpatient and inpatient cohorts were merged here to yield a higher support for each condition; however split performances are available on Table S8. Similarly, per-study performances are available on Table S9. Overall, the pipeline reached a macro average F1-score, PPV, sensitivity, specificity of 95.6, 95.3, 95.9, and 99.2, respectively. Additionally, entity-level metrics for this pipeline are available on Table S6. Comparing stay-level metrics with entity-level metrics shows the importance of the aggregation module, since it improved macro average F1-score, PPV, and sensitivity by 4.7, 2.4, and 6.7 and points, respectively. The performance of the isolated qualification module was assessed in Table S7, also showing the superiority of the ML approach.

**Table 2.** Stay-level performances (F1-score, positive predictive value, sensitivity, and specificity) of the main NLP-ML-CLINICAL pipeline used to predict 18 conditions from clinical notes considering inpatient and outpatient stays of the 3 disease-specific cohorts.

		F1	Positive predictive value	Sensitivity	Specificity
Myocardial infarction		92.7 (87.1-96.5)	92.7 (85.7-97.6)	92.7 (85.3-97.7)	98.8 (97.7-99.6)
Congestive heart failure		96.3 (92.4-98.2)	96.3 (92.7-98.8)	96.3 (91.1-98.8)	98.6 (97.3-99.5)
Peripheral vascular disease		97.6 (96.3-98.8)	97.6 (96.0-99.2)	97.6 (95.2-99.2)	98.3 (97.2-99.4)
Cerebrovascular disease		94.1 (89.2-97.2)	91.4 (83.3-97.2)	97.0 (90.9-97.2)	98.9 (97.8-99.6)
Dementia		93.5 (88.1-96.9)	90.6 (81.8-96.9)	96.7 (90.3-97.0)	98.9 (97.8-99.6)
Chronic pulmonary disease		94.1 (90.4-97.6)	91.8 (85.2-98.4)	96.6 (91.2-98.3)	97.9 (96.3-99.6)
Rheumatologic disease		92.1 (82.7-97.0)	96.7 (85.6-97.0)	87.9 (79.2-97.0)	99.6 (98.5-99.6)
Peptic ulcer disease^a^		100.0 (-)	100.0 (-)	100.0 (-)	100.0 (-)
Liver^a^	*Mild*	100.0 (-)	100.0 (-)	100.0 (-)	100.0 (-)
	*Moderate to severe*	100.0 (-)	100.0 (-)	100.0 (-)	100.0 (-)
	*Any*	100.0 (-)	100.0 (-)	100.0 (-)	100.0 (-)
Diabetes^a^	*Without complications*	96.6 (94.0-98.9)	100.0 (-)	93.3 (88.7-97.8)	100.0 (-)
	*With complications*	93.3 (85.7-96.8)	87.5 (75.0-93.8)	100.0 (-)	99.3 (98.6-99.6)
	*Any*	99.1 (97.4-99.2)	100.0 (-)	98.3 (94.9-98.3)	100.0 (-)
Hemiplegia^b^		–	89.3 (82.4-96.4)	–	–
Renal disease		85.0 (75.5-93.1)	85.0 (71.4-95.2)	85.0 (66.6-95.2)	98.9 (97.9-99.6)
Solid tumor^a^	*Localized*	93.1 (89.8-97.1)	92.2 (86.2-98.1)	94.0 (88.0-98.0)	98.4 (97.2-99.6)
	*Metastatic*	97.1 (90.7-97.3)	100.0 (-)	94.4 (83.1-94.7)	100.0 (-)
	*Any*	95.6 (91.6-97.9)	95.6 (88.2-98.6)	95.6 (91.4-98.6)	98.7 (96.6-99.6)
Leukemia^a^		96.3 (87.9-96.6)	92.9 (78.4-93.3)	100.0 (-)	99.7 (99.0-99.7)
Lymphoma^a^		97.6 (92.2-97.7)	100.0 (-)	95.2 (85.6-95.5)	100.0 (-)
AIDS^b^		–	60.4 (51.7-73.6)	–	–
Alcohol consumption^a^	*Present*	84.2 (62.3-90.1)	72.7 (45.2-82.0)	100.0 (-)	99.0 (98.0-99.3)
	*Stopped*	80.0 (50.0-94.1)	100.0 (-)	66.7 (33.3-88.9)	100.0 (-)
	*Any*	100.0 (-)	100.0 (-)	100.0 (-)	100.0 (-)
Tobacco consumption	*Present*	78.0 (64.8-86.5)	76.2 (57.1-95.0)	80.0 (64.8-94.1)	98.2 (96.8-99.6)
	*Stopped*	93.9 (87.1-97.1)	93.9 (82.3-97.1)	93.9 (87.5-97.1)	99.3 (97.8-99.6)
	*Any*	95.3 (91.6-97.3)	94.4 (87.5-98.1)	96.2 (90.9-98.1)	98.8 (97.2-99.6)
Total	Micro average	95.7 (94.5-96.3)	95.4 (94.0-96.3)	96.0 (94.0-96.7)	99.2 (99.0-99.4)
	Macro average	95.6 (94.0-96.2)	95.3 (93.9-96.1)	95.9 (93.6-96.7)	99.2 (98.9-99.4)
	Weighted average	95.7 (94.5-96.3)	95.5 (94.2-96.3)	96.0 (94.0-96.7)	98.9 (98.6-99.2)

95% confidence intervals were estimated by bootstrapping.

a In case of metrics at 100, no bootstraping was performed.

b For these conditions, only the positive predictive value was computed.

Table 3 compares the stay-level performances of the NLP-ML-CLINICAL pipeline with our 3 alternative pipelines, namely NLP-ML-GENERIC, NLP-ML-RB, and CLAIM, considering only inpatient stays. Overall, the F1-score, PPV, and sensitivity were the highest for the NLP-ML-CLINICAL pipeline whereas the CLAIM pipeline showed the highest specificity.

**Table 3.** Stay-level performances of the main NLP-ML-CLINICAL pipeline compared to 3 alternative pipelines based on alternative technologies NLP-ML-GENERIC, NLP-RB, and CLAIM. Performances are computed on the inpatient stays of the 3 cohorts. For each condition and metric, value of best performing pipeline is represented in bold. The 95% confidence intervals of the averaged metrics were computed by bootstrapping.

	F1				Positive predictive value				Sensitivity				Specificity			
	NLP-ML-CLINICAL	NLP-ML-GENERIC	NLP-RB	CLAIM	NLP-ML-CLINICAL	NLP-ML-GENERIC	NLP-RB	CLAIM	NLP-ML-CLINICAL	NLP-ML-GENERIC	NLP-RB	CLAIM	NLP-ML-CLINICAL	NLP-ML-GENERIC	NLP-RB	CLAIM
Myocardial infarction	**94.9**	93.3	84.8	55.8	**90.3**	87.5	73.7	80.0	**100.0**	**100.0**	**100.0**	42.9	97.5	96.7	91.8	**98.9**
Congestive heart failure	**95.7**	**95.7**	76.8	75.2	**94.9**	**94.9**	62.4	88.4	96.6	96.6	**100.0**	65.5	96.7	96.7	62.0	**97.9**
Peripheral vascular disease	**97.2**	96.6	88.0	50.8	**97.8**	97.7	80.0	82.5	96.7	95.6	**97.8**	36.7	**96.7**	**96.7**	63.3	**96.7**
Cerebrovascular disease	**96.0**	92.3	81.4	43.8	**92.3**	85.7	68.6	87.5	**100.0**	**100.0**	**100.0**	29.2	98.4	96.8	91.3	**99.6**
Dementia	**92.3**	88.5	90.6	57.9	**88.9**	85.2	85.7	84.6	**96.0**	92.0	**96.0**	44.0	97.6	96.8	96.8	**99.3**
Chronic pulmonary disease	**92.8**	91.2	82.1	44.9	**88.9**	88.6	71.1	68.8	**97.0**	93.9	**97.0**	33.3	96.6	96.6	88.9	**98.1**
Rheumatologic disease	**87.5**	**87.5**	78.9	69.2	93.3	93.3	71.4	**100.0**	82.4	82.4	**88.2**	52.9	99.2	99.2	95.5	**100.0**
Peptic ulcer disease	100.0	100.0	91.7	–	**100.0**	**100.0**	84.6	0.0	**100.0**	**100.0**	**100.0**	0.0	**100.0**	**100.0**	98.6	99.3
Liver disease	**100.0**	**100.0**	94.7	80.0	**100.0**	**100.0**	90.0	**100.0**	**100.0**	**100.0**	**100.0**	66.7	**100.0**	**100.0**	99.3	**100.0**
Diabetes	**98.6**	95.9	90.2	65.5	**100.0**	97.2	82.2	90.5	97.3	94.6	**100.0**	51.4	**100.0**	99.1	92.9	99.2
Hemiplegia^a^	**-**	**-**	–	–	**82.8**	**82.8**	72.7	77.8	**-**	**-**	**-**	–	–	–	–	**-**
Renal disease	**85.7**	**85.7**	80.0	8.3	**92.3**	**92.3**	80.0	11.1	**80.0**	**80.0**	**80.0**	6.7	**99.3**	**99.3**	97.8	97.2
Solid tumor	**97.2**	**97.2**	83.3	64.6	**94.6**	**94.6**	71.4	70.0	**100.0**	**100.0**	**100.0**	60.0	**98.3**	**98.3**	87.8	96.6
Leukemia	94.1	**100.0**	88.9	70.6	88.9	**100.0**	80.0	66.7	**100.0**	**100.0**	**100.0**	75.0	99.3	**100.0**	98.6	99.0
Lymphoma	**95.7**	**95.7**	68.8	55.6	**100.0**	**100.0**	55.0	83.3	**91.7**	**91.7**	**91.7**	41.7	**100.0**	**100.0**	93.5	99.7
AIDS^a^	–	**-**	–	–	55.6	68.2	48.6	**80.0**	–	–	**-**	–	–	–	–	**-**
Alcohol consumption	**100.0**	**100.0**	49.1	44.4	**100.0**	**100.0**	32.6	**100.0**	**100.0**	**100.0**	**100.0**	28.6	**100.0**	**100.0**	78.7	**100.0**
Tobacco consumption	**94.3**	93.0	68.0	24.4	**94.3**	91.7	52.3	83.3	94.3	94.3	**97.1**	14.3	98.3	97.4	73.0	**99.6**
Micro average	**95.5 (93.8-95.9)**	94.5 (92.4-95.1)	80.7 (78.7-83.5)	54.7 (50.2-57.8)	**94.8 (92.8-95.6)**	93.7 (91.4-94.4)	68.8 (66.1-73.0)	79.3 (73.6-82.4)	96.2 (94.2-96.9)	95.3 (93.2-96.1)	**97.6 (96.4-98.2)**	41.7 (37.3-45.7)	98.8 (98.3-99.0)	98.5 (98.0-98.7)	89.7 (89.0-90.9)	**98.9 (98.5-99.0)**
Macro average	**95.1 (92.7-95.7)**	94.5 (92.1-95.2)	81.1 (78.2-83.4)	54.1 (48.4-57.9)	**94.8 (92.2-95.3)**	94.3 (91.5-95.2)	71.3 (68.3-75.0)	74.8 (70.5-77.3)	95.7 (93.2-96.4)	95.1 (92.7-95.9)	**96.7 (95.0-97.8)**	40.5 (35.3-43.9)	98.6 (98.1-98.9)	98.4 (97.8-98.6)	88.1 (87.3-89.5)	**98.8 (98.4-99.0)**
Weighted average	**95.5 (93.7-95.9)**	94.5 (92.5-95.1)	81.9 (80.0-84.7)	53.0 (48.7-56.5)	**94.9 (93.1-95.7)**	93.9 (91.8-94.6)	71.6 (69.1-76.0)	79.1 (75.6-81.9)	96.2 (94.2-96.9)	95.3 (93.2-96.1)	**97.6 (96.4-98.2)**	41.7 (37.3-45.7)	98.0 (97.2-98.5)	97.7 (96.6-98.2)	81.1 (79.8-83.8)	**98.3 (97.8-98.6)**

a For these conditions, only the positive predictive value was computed.

**Figure 3.**
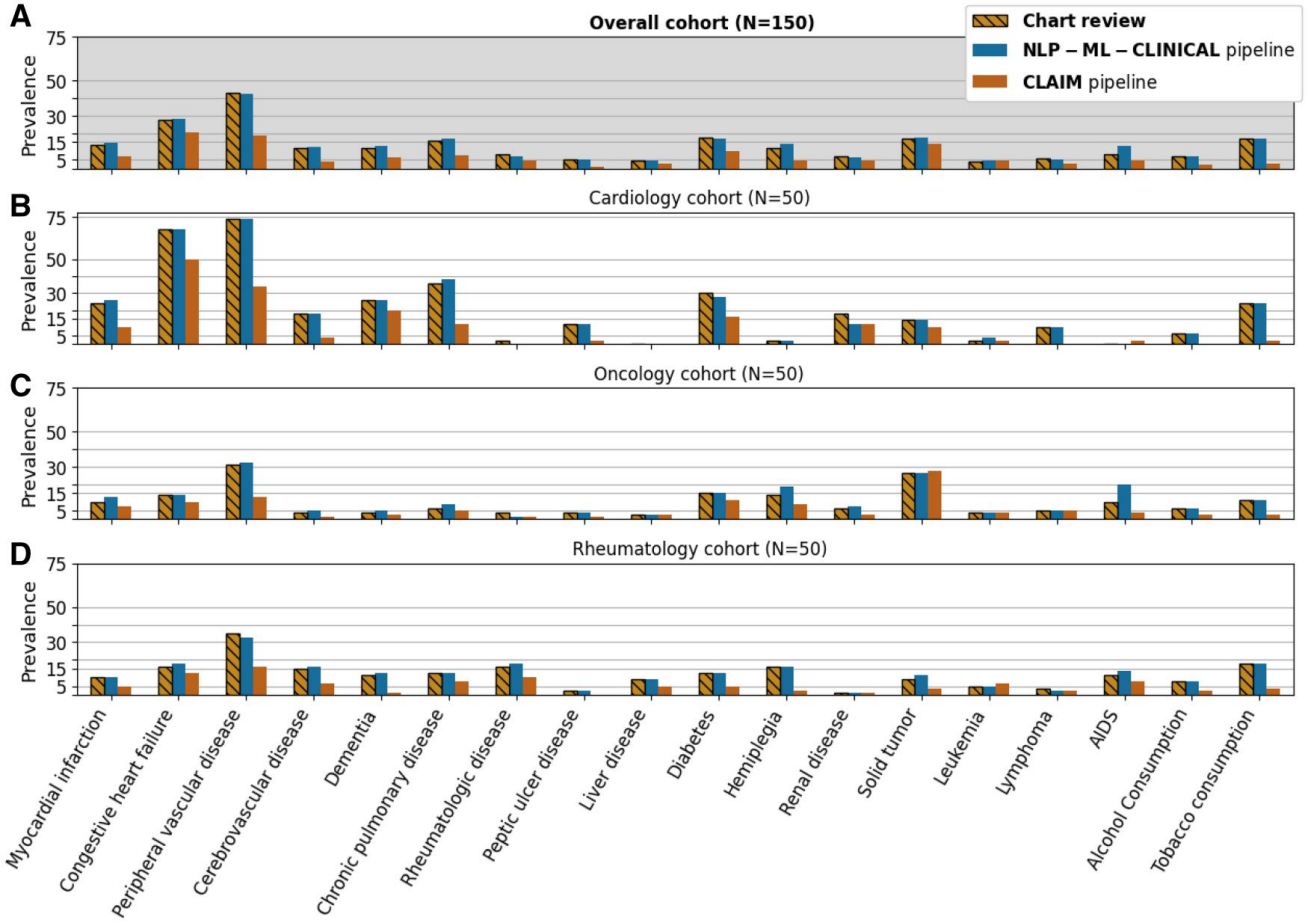
Prevalence of conditions estimated either via the main NLP-ML-CLINICAL pipeline (blue), the alternative CLAIM pipeline (orange), or a chart review of clinical notes (hashed yellow) considering inpatient stays of the cardiology (B), oncology (C), rheumatology (D), and overall (A) cohorts, respectively (number of stays in brackets).

The prevalence of each condition computed on this same cohort by either the NLP-ML-CLINICAL and CLAIM pipeline was compared to the chart review prevalence (see Figure 3). Prevalences computed with NLP-ML-CLINICAL and CLAIM differed from chart review prevalences by 0.82 points and 8.1 points on average, respectively.

**Figure 4.**
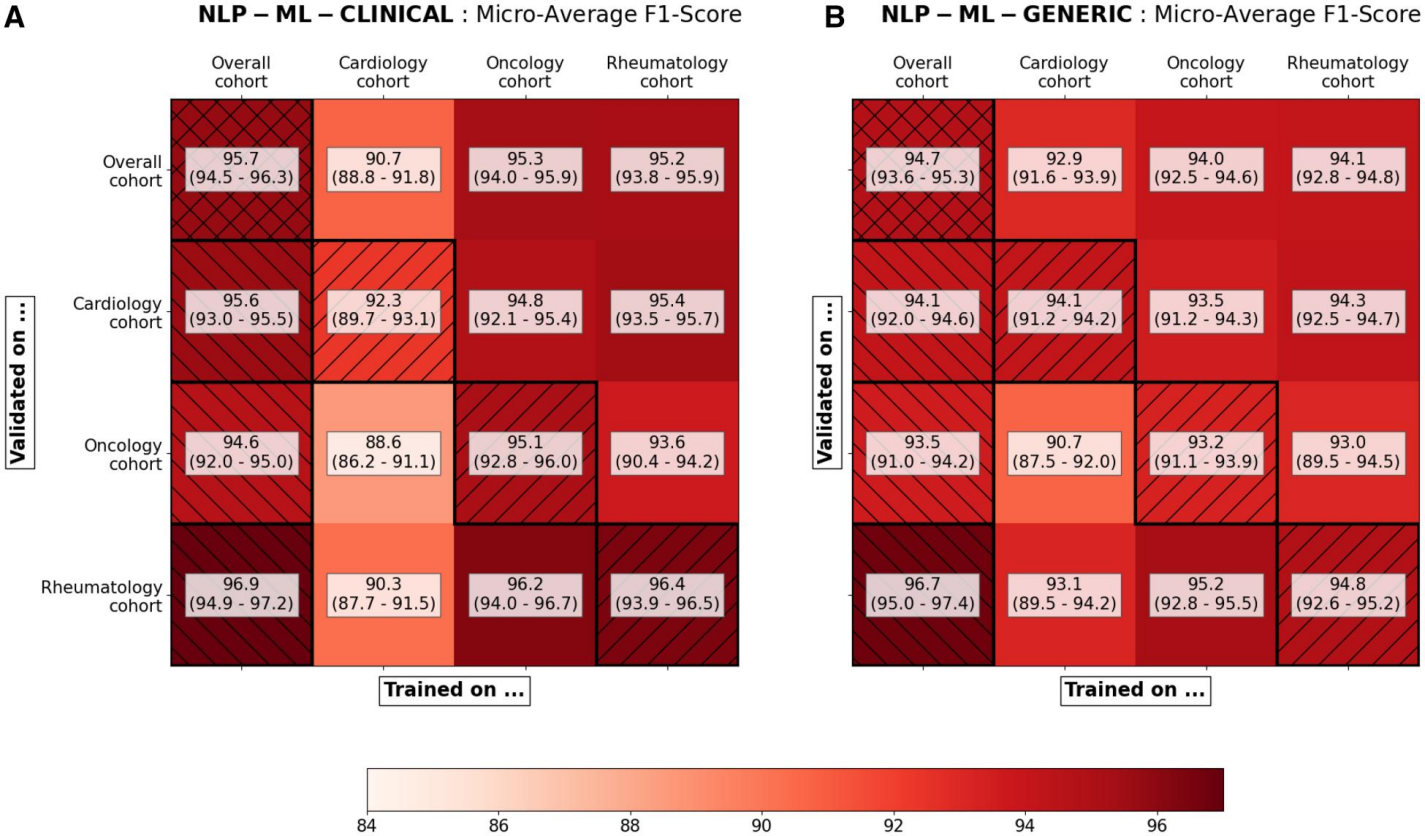
Comparison of the stay-level micro-averaged F1-score of the pipelines and their 95% confidence intervals simulating various levels of collaboration. (A) The main NLP-ML-CLINICAL pipeline that reuses the clinical language model of a former project is compared to (B) the NLP-ML-GENERIC pipeline that only uses a public language model. In each case, the performances are estimated on every (training study, validation study) couple available. The first column (left hatched) shows performances of the final pipeline trained on the 3 cohorts together. The main diagonal (right hatched) shows performances of the final pipeline in a hypothetical non-collaborative setting, ie, trained and validated on the same cohort. 95% confidence intervals were estimated by bootstrapping.

Figure 4 compares different collaborative settings, showing the evolution of the F1-score when training and validating either NLP-ML-CLINICAL (left) or NLP-ML-GENERIC (right) on each (training cohort, validation cohort) couple. In each of these 2 cases, the first column shows the F1-score of the pipeline trained on the overall cohort, and the principal diagonal shows the F1-score of the pipeline in a hypothetical less collaborative setting, limiting both the training and validation data to each study’s cohort. In accordance with Table 3, in 75% of all cases NLP-ML-CLINICAL was better than NLP-ML-GENERIC, thus showing an average improvement related to re-using a clinical language model shared through a secure model hub. Regarding NLP-ML-CLINICAL, training on the overall cohort outperformed training on any other study cohort, showing the improvement provided to the annotation of the common training dataset by increasing the collaboration among the 3 cohort studies. When validating on a specific cohort, results vary and either the pipeline trained on the same cohort as the validation cohort (oncology study) or the pipeline trained on the overall cohort (cardiology and rheumatology cohort) is best performing. As shown in Table S4, privacy exposure is greatly reduced using our workflow compared to a simpler collaborative workflow that would take place in a single environment accessed by all the clinicians and data scientists.

Figure S5 compares the CCI computed using NLP-ML-CLINICAL with either the CLAIM pipeline or CCIs computed at the point of care. Strong discrepancies are observed.

## Discussion

We developed, validated, and shared a hybrid NLP pipeline to efficiently detect conditions mentioned in clinical notes written in French. We quantified the added value of NLP (compared to claim-based) approaches, and of advanced (compared to simpler) NLP technologies. Moreover, we measured the performance increase obtained following a collaborative setting that leveraged the hybrid architecture of the pipeline to enable the association of various medical experts, while complying with data access restrictions enforced on a CDW.

The performances of our main NLP-ML-CLINICAL pipeline are comparable to the best published results regarding similar approaches, although slightly lower than those reached by Singh et al^13^^,^^15^^,^^37^ We emphasize that these studies remain difficult to compare as they focus on different languages (French vs English) and texts (eg, Singh et al consider only medical and surgical history sections). Aggregating extractions from the entity level to the stay level significantly improves performances, which is a known and notable result as stay-level or patient-level features are often of higher importance and interest than entity-level features, eg, in epidemiological studies.^15^ Notably, sensitivity is greatly improved, as the aggregation step allows for missed entities of a specific condition to be compensated by other occurrences of the same condition. Similarly, conditions with 2 levels of severity benefit from this aggregation since severity would not necessarily be mentioned on each entity. For instance, a patient’s diabetes will be mentioned as complicated, eg, in the initial section of the clinical notes, and then referred to simply as the patient’s diabetes. Thus, considering entire notes without restrictions to specific sections proved to be beneficial.

Some conditions proved to be more challenging to extract, either because their mentions vary greatly, or because they require an additional disambiguation task. Some examples include: correctly classifying the current status of alcohol or tobacco consumption (current or stopped) with a rule-based approach was hindered by the highly diverse formulations. The stage of renal failure was often not explicitly mentioned but could be implicit from the context or inferred from other information in the clinical note. AIDS was sometimes mentioned *as is* in the text, but most often it was mentioned as HIV+ along with an opportunistic disease, making it harder to discriminate. Heart failure could often refer to an acute symptom and was difficult to distinguish from a chronic problem.

In accordance with the literature, the superiority of NLP-ML-CLINICAL over NLP-ML-GENERIC supports the interest of using large language models trained on clinical corpora, although their privacy-sensitive nature makes them more difficult to share.^21^^,^^38^ More generally, it shows the superiority of ML-based pipelines over simpler rule-based and claim-based pipelines, the latter especially struggling with a poor sensitivity. In fact, we showed that hybrid pipelines could both outperform claim-based pipelines when relevant (eg, on inpatient stays) and replace it when no claim data is available (eg, on outpatient stays). Claim-based pipeline still outperforms each NLP pipeline regarding specificity: claim data suffers from systemic undercoding, especially with conditions that have little to no impact on the stay’s billing such as alcohol and tobacco consumption, hypertension or asthma. This implies a low sensitivity, jointly with a high specificity.

We demonstrated the relevance of developing a common pipeline that supports various disease-specific studies conducted on routine data. In fact, both overall performance and generalizability were increased by the collaborative setting: as this setting allows for a larger training set along with more diverse training examples, these gains were expected. Additionally, what couldn’t be measured directly but certainly had a significant impact was the importance of collaboration on the rule-based NER modules: Concertation meetings showed the complexity of building a robust and clinically relevant terminology, and working with multiple clinicians from various specialties most certainly helped to converge toward this goal.

Our work reflects the evolution of privacy protection. Historically, datasets were minimized and de-identified and, once deemed non-sensitive, released to their end users for analysis. Many studies have demonstrated that this solution was no longer applicable in the era of artificial intelligence (AI), as those models often require accessing unminimized and high-dimensional datasets that cannot be anonymized.^4^^,^^20^^,^^39^ Indeed, anonymizing high dimensional structured data is usually associated with the destruction of the statistical patterns of interest^40^ and, although NLP algorithms may suppress most of the names, dates of birth, etc. mentioned in texts, those data cannot be fully anonymized using an algorithm.^41^^,^^42^ To address this issue, many call for a switch from an *anonymize-and-release* to a *privacy-through-security* paradigm.^39^^,^^43^ Secured platforms such as CDWs should host data and models that remain sensitive, while finely controlling accesses and queries. New technologies have been proposed to this end such as federated learning and differential privacy.^44^^,^^45^ However, none of these technologies provides a silver bullet solution yet, and obtaining privacy-conscientious workflows still requires combining various security measures in architectures adapted to specific uses.^39^^,^^43^ In this study, we combined access-control, data minimization, and model securing in a workflow adapted to the training and validation of NLP pipelines dedicated to the detection of medical conditions. In particular, we leveraged the modularity of our hybrid pipelines to distinguish 3 stages. First, the rule-based NER modules, that were not privacy-sensitive, were shared without constraints among the 3 cohort-specific environments and iteratively improved by clinicians. Second, the data scientist worked in a dedicated environment to train the qualification module. He accessed only the annotated dataset and a pre-trained clinical language. Third, the trained qualification module was shared back with disease-specific environments for the clinicians to validate the pipelines. For the second and third stages, the storage and re-use of potentially privacy-sensitive ML models was made possible using a dedicated hub whose access required a prior IRB validation.^5^^,^^29^

Our study has limitations. First, inter-annotator agreement could not be measured, as each investigator accessed only her disease-specific cohort. Also, using a pre-annotation step before manual annotation might lead to a confirmation bias.^46^ Nevertheless, it substantially speeds up the annotation and enables the production of higher volumes of annotated data, which arguably increases the robustness of the process. Second, although different technologies were explored, we focused for this comparison on the qualification module and always used a simple aggregation rule to define variables at the stay level. Implementing a more advanced aggregation module may improve the overall pipeline’s performance. We also separately analyzed either clinical notes or claim data: jointly considering text and additional variables such as laboratory test results, drugs or claim data may improve the performances.^7^ The ML-based qualification module could also be extended to classify, if relevant, the status of the condition, since this task can be complex and highly dependent on context. Third, the privacy-enhancing workflow we propose is a single step in the direction of efficient and privacy-conscientious architectures as (1) data exposition, although reduced, remains non-negligible, (2) this workflow does not apply to other tasks such as data exploration, which is a cornerstone of data analysis, and (3) it relies on the prior integration of data in a common technical and regulatory platform such as a CDW and consequently does not address the central issue of model sharing across organizations.

## Conclusion

We followed a new collaborative and privacy-enhancing workflow to develop and validate a hybrid rule-based/ML pipeline dedicated to the automatic detection of common conditions in clinical notes written in French. Thus, we demonstrated that using advanced NLP technologies led to higher performances when compared to classical approaches and that hybrid architectures could be leveraged to associate various experts working on a common CDW. This work is a first step in the direction of better adapting data analysis workflows both to emerging ML technologies and to the regulatory constraints. We hope that this work will pave the way to closer collaborations among a wide community of clinicians, investigators and engineers working on CDWs.

## Supplementary Material

ocae069_Supplementary_Data

## Data Availability

The code for the hybrid model and the experiments of this study were made available on a dedicated GitHub repository.^47^ As mentioned in the study, the NER modules were made available in the 0.8.1 release of the EDS-NLP library and the trained qualification module was made available into AP-HP’s CDW through a secure registry.^34^
