# Supplementary material for: Collaborative and privacy-enhancing workflows on a clinical data warehouse: an example developing natural language processing pipelines to detect medical conditions
Source: J Am Med Inform Assoc. 2024 Apr 4;31(6):1280–90. doi: 10.1093/jamia/ocae069 (PMC11105139; doi:10.1093/jamia/ocae069)
Supplement: ocae069_Supplementary_Data [file ocae069_supplementary_data.zip › ocae069_Supplementary_Data/sm_bis_final.pdf]

1 Appendix A: Description of the cardiology, rheumatology and oncology cohorts

1.1 Per-cohort inclusion criteria

1.1.1 Oncology cohort

The cohort is composed of 337670 patients with a diagnosis of cancer. Characterization is done via diagnosis claim codes (*International Classification of Diseases, 10th revision* -ICD-10-), clinical procedure codes (French *Classification Commune des Actes Médicaux* -CCAM-) or diagnosis related group codes (French *Groupement Homogène de Malades* -GHM-) attached to a hospital stay occurring between 2006 and 2020 (Supplementary Table S1 and [1]).

E.K. was the clinician studying this cohort's data. She was a PhD student in data science at the time of this study and was thus familiar with the field of NLP applied to clinical notes.

ICD-10 Codes	CCAM Codes	GHM Codes
C00-C97, D00-D09, D37-D48, B21, D630, E883, G533, G550, G631, G732, G941, J700, J701, K520, K627, L412, L580, L581, L598, L599, M360, M361, M906, M907, M962, M965, N304, O356, T451, T66, T860, Z08, Z510, Z511, Z9480, Y431, Y432, Y433, Y632, Z85, Z923, Z926	YYYY042, ZZNL065, ZZML001, ZZNL063, FEFF002, ZZML003, ZZNL054, ZZMK018, ZZMP017, ZZNL051, HLNM001, BGLA002, HLNK001, ZZNL050, EDLF017, ZZML002, HKFA007, ZZNL048, AFLB003, ZZNL062, ZZMP015, ZZLF900, EDLF014, EDLF016, ZZMK020, ZZNL053, FDFB001, FELF009, FELF010, GGLB001, ZZNL061, GENE003, HHGE010, KCNL004, ZZMK011, ZZNL058, HELE001, EDLF015, ZZLF004, ZZMP012, ZANL001, ZZNL059, ZZNL064, AGMP001, EDLL002, FEFF001, GGLB008, HLNN900, HPLA002, JDLA001, JDLA001, JLNL005, ZZMK002, ZZMK013, ZZMK024, ZZMP016, ZZNL003, ZZNL005, ZZNL016, ZZNL016, ZZNL018, ZZNL019, ZZNL047, ZZNL060	01M261, 01M262, 01M263, 01M264, 01M26T, 03M071, 03M072, 03M073, 03M074, 03M07T, 06C161, 06C162, 06C163, 06C164, 06M051, 06M052, 06M053, 06M054, 06M05T, 06M131, 06M132, 06M133, 06M134, 06M13T, 07C061, 07C062, 07C063, 07C064, 07C091, 07C092, 07C093, 07C094, 07M061, 07M062, 07M063, 07M064, 07M06T, 08C291, 08C292, 08C293, 08C294, 08C29J, 08M241, 08M242, 08M243, 08M244, 08M24T, 09C041, 09C042, 09C043, 09C044, 09C051, 09C052, 09C053, 09C054, 09C05J, 09M101, 09M102, 09M103, 09M104, 09M10T, 10C111, 10C112, 10C113, 10C114, 12C051, 12C052, 12C053, 12C054, 12C091, 12C092, 12C093, 12C094, 12C111, 12C112, 12C113, 12C114, 12M031, 12M032, 12M033, 12M034, 12M03T, 13C051, 13C052, 13C053, 13C054, 13C111, 13C112, 13C113, 13C114, 13C11J, 13C141, 13C142, 13C143, 13C144, 13M031, 13M032, 13M033, 13M034, 13M03T, 17C021, 17C022, 17C023, 17C024, 17C031, 17C032, 17C033, 17C034, 17C03J, 17M051, 17M052, 17M053, 17M054, 17M061, 17M062, 17M063, 17M064, 17M06T, 17M081, 17M082, 17M083, 17M084, 17M08T, 17M091, 17M092, 17M093, 17M094, 17M09T, 17M111, 17M112, 17M113, 17M114, 17M11T, 17M121, 17M122, 17M123, 17M124, 17M12T, 28Z07Z, 28Z10Z, 28Z11Z, 28Z18Z, 28Z19Z, 28Z20Z, 28Z21Z, 28Z22Z, 28Z23Z, 28Z24Z, 28Z25Z, 27Z021, 27Z022, 27Z023, 27Z024, 27Z03Z, 27Z04J

Table S1: Codes used to select the oncology cohort

1.1.2 Cardiology cohort

The cohort is composed of 11395 patients aged 75 or more, hospitalized after August 2017 in a cardiology, pneumology or geriatry department and presenting either a diagnosis of acute cardiac failure, pulmonary embolism, pulmonary infection or chronic pulmonary obstructive disease (extraction via diagnosis claim codes), and a dosage of natriuretic peptides. Biological Characterization is done via diagnosis claim codes and laboratory test results (*Logical Observation Identifiers Names and Codes -LOINC-*). See Supplementary Table S2.

E.B. was the clinician studying this cohort's data.

ICD-10 Codes	LOINC Codes
I50, I110, I26, J441, J209, J46, J12, J13, J14, J15, J16, J17, J18, J40, J41, J42, J43, J44, J45, J47, J20	33762-6, 30934-4

Table S2: ICD-10 diagnostic codes and LOINC laboratory codes used to select the cardiology cohort

1.1.3 Rheumatology cohort

The cohort is composed of 38384 patients aged 15 or older with at least one hospital stay between January 7th, 2017 and December 31st, 2020 with a medical history of either systemic lupus erythematosus, Takayasu disease, scleroderma or antiphospholipid syndrome. Characterization is done via diagnosis claim codes and verbatim mentions of these diseases in clinical reports. See Supplementary Table S3.

C.G. was the clinician studying this cohort's data. As E.K., she was a PhD student in data science at the time of this study and was thus familiar with the field of NLP applied to clinical notes.

Disease	ICD-10 Codes	Keyword(s) (*French*)
Lupus	M310, M321, M328, M329, L930, L931	”lupus”
Takayasu disease	M314	”Takayasu”
Sclerodermia	M340, M341, M348, M349	”sclerodermie”, ”CREST”
Antiphospholipid antibody syndrome	D686	”SAPL”, ”syndrome des antiphospholipides”, ”CAPS”

Table S3: ICD-10 diagnostic codes and keywords used to select the rheumatology cohort.

1.2 Training and validation datasets

To split the datasets of the different projects in three development datasets and three validation datasets while avoiding data leakage between these sub-cohorts, we would ideally need to share patient identities. Yet, identity linkage between the different isolated environments is impossible due to privacy issues. We therefore use the recently published technique of fold-stratified (cross)-validation, a privacy-preserving technique that avoids patient-level linkage while enabling splitting the datasets in development datasets and validation datasets.[2] In order to apply this technique, we stratify the cohorts using a covariate that is shared among pseudonymized records, namely the birth date. By ensuring that every patient with a specific birth date can only be found in a single sub-cohort, we guaranteed that two digital identities corresponding to the same patient couldn't be found simultaneously in two distinct sub-cohorts.

Each cohort was split into three sub-cohorts. As shown on the flowchart (Supplementary Figure S1), we used this number along with a hash function to

1. Stratify patients and avoid multiple annotations of the same visits: to do so, project n°i worked with patients which birthday BD verifies $Hash(BD) \equiv i \pmod 3$ with $Hash$ being the MurmurHash3 function.
2. Further split patients into training sets and a validation set in each project: if $Hash(Hash(BD)) \equiv j \pmod 2$, the corresponding patient would be put into the dataset for the j-th iteration. Patients with $j = 0$ were included in the validation dataset.

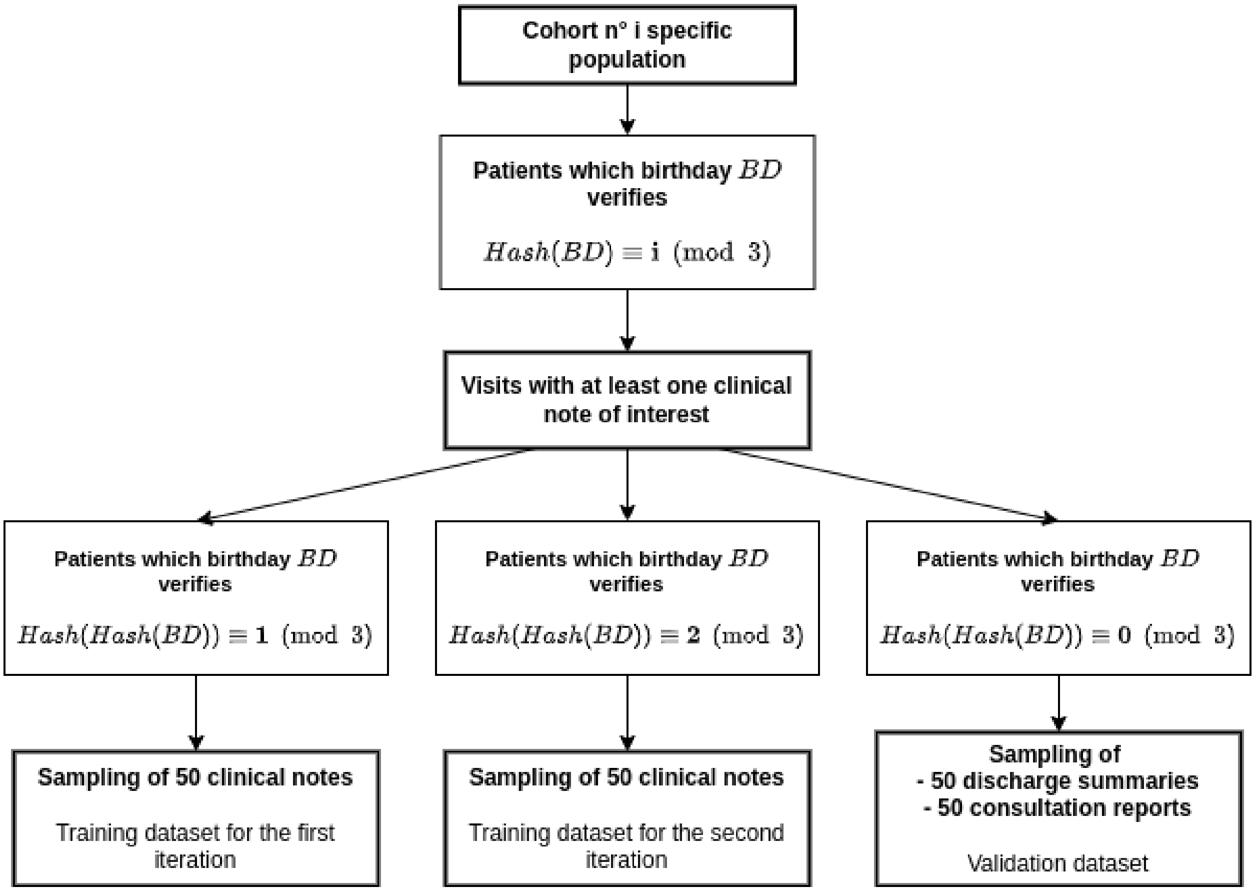

Figure S1: Inclusion flowchart for one study guaranteeing the absence of data leakage among studies

2 Appendix B: Details on the pipelines

2.1 Natural language processing pipeline

2.1.1 Preprocessing

The **preprocessing** module splits the raw text into tokens (individual words) and sentences. It then normalizes the text by

- switching case to lowercase (*Diabète* et *'Behçet'* → *diabète* et *'behçet'*)
- removing diacritics (*diabète* et *'behçet'* → *diabete* et *'behcet'*)
- uniforming quotes and apostrophes (*diabete* et *'behcet'* → *diabete* et *"behcet"*)

Finally, it detects frequent pollution patterns in texts which were identified as potential sources of false positives for the named entity recognition (NER) modules. For instance, it will tag e-mail address and websites URLs (e.g., to avoid matching patterns such as `www.diabetes.fr` or `accueil.vascularites@aphp.fr`) or parsed laboratory results, which often contain disease names (moreover, the exploitation of such biology results is outside of this article's scope).

2.1.2 NER modules

For each comorbidity, the NER module consists of a set of *matching dictionaries*. A *matching dictionary* has three main components which are called sequentially in the module (see Supplementary Figure S2 for an example of NER module structure).

The first component is the *matching component*. Using a set of regular expressions, it will find *anchors* (i.e., raw mentions of a specific disease or condition) in text. The second component is the *exclusion component*. For each *anchor*, it applies exclusion rules on its surrounding window to remove incorrect or undesirable matches. The third component is the *refining component*. Like the *exclusion component*, it will search in each *anchor*'s surrounding window to extract additional information that will help further discriminate the match (for instance to set, if applicable, the status or severity level).

2.1.3 Qualification module

The **qualification module** characterizes the context of each occurrence. The simple detection of an occurrence of a concept in clinical notes is often misleading, as an occurrence of a term may for instance be negated, formulated as a hypothesis or related to a member of the family. Although the paper's main pipeline uses a qualifier based on Machine Learning (ML), both a **rule-based approach** and an **ML-based approach** were carried out.

The rule-based module (Supplementary Figure S3, left) uses a list of regular expression names *cues* to detect marks of negation, hypothesis or familial context. By linking those *cues* to extracted conditions, we flagged each condition as being either

- negated: Is the detected condition negated in the text ?
- family-related: Is the detected condition referring to a member of the patient instead of the patient himself ?
- hypothetical: Is there a hypothesis marker ?

This rule-based module was not developed as part of this study. It was implemented beforehand in the EDS-NLP library and adapts the NegEx algorithm to the french language.[3,4].

Finally, a unique boolean flag is constructed by taking the boolean OR of those three flags.

Instead of tagging multiple modalities like the rule-based qualification module, for this ML approach we trained a neural network to classify an extraction as valid or not, encapsulating the qualification in a single boolean variable (Supplementary Figure S3, right). For the classification head, a mean pooler layer was used to average all embedding vectors from an entity into a single vector. Following was a fully connected layer. This architecture allowed us to handle the qualification task as a sentence classification task, assigning one boolean label to each snippet.

2.1.4 Stay-level classification

The **stay-level aggregation module** aims at reconciling, for each concept, extractions from a single note by doing a rule-based aggregation. The goal is then to end up with stay-level labels by using associated notes. We used a simple but efficient rule that considered, for each condition, the set of all related entities which were not discarded by the qualification module. If this set was not empty, we considered the condition as present. Moreover, in the case of conditions with multiple statuses, we kept the status of the most severe entity from the set.

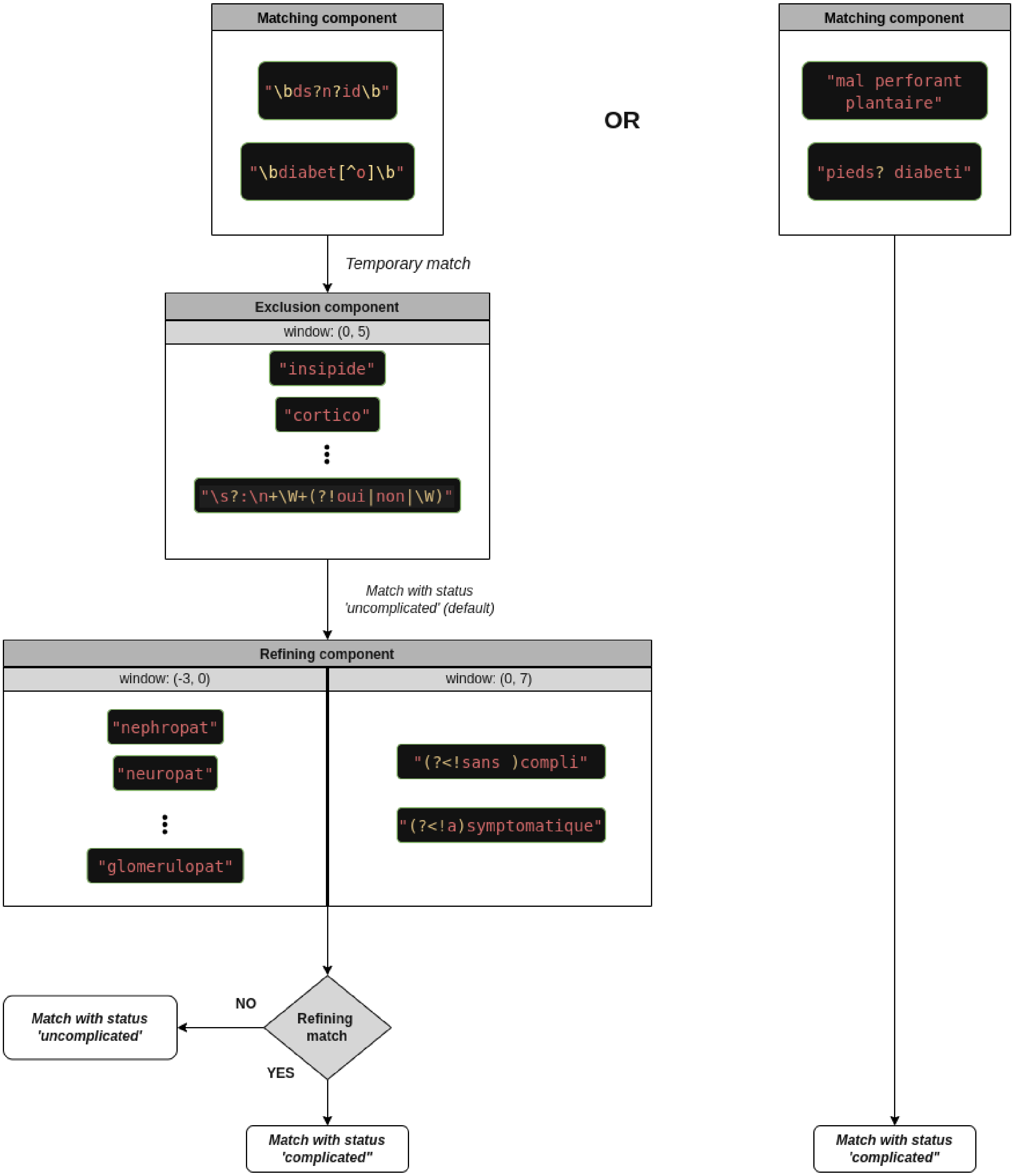

Figure S2: Illustration of a named entity recognition algorithm considering the example of *Diabetes*. Only subsets of patterns are shown in each component.

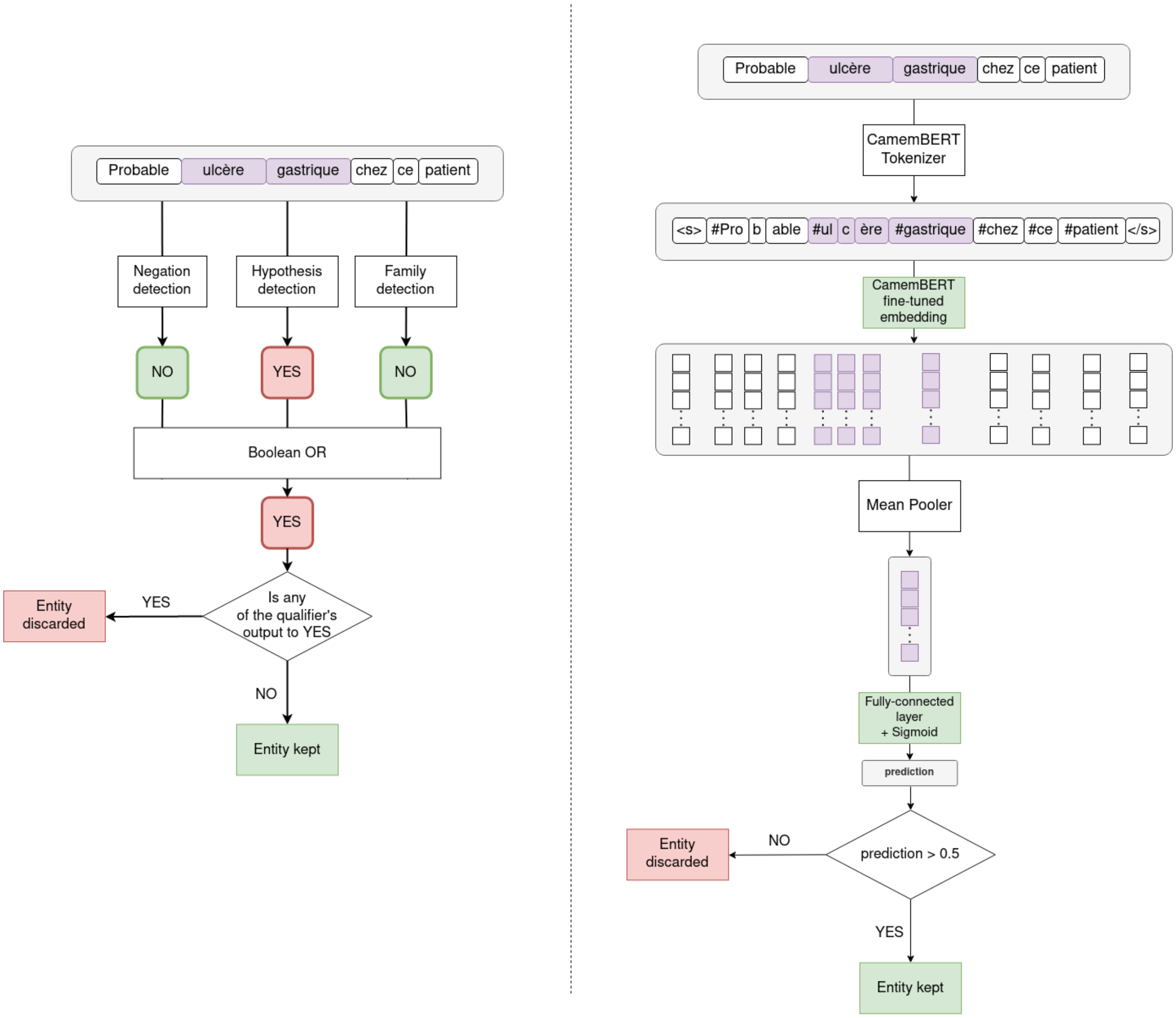

Figure S3: Structure of the rule-based (left) and machine learning (ML)-based (right) qualification modules. Regarding the ML-based module, components whose parameters were tuned during the training procedure are shown in green.

3 Appendix C: Pipelines development

3.1 Notes sampling

We upsampled the notes used to train the Qualification module in order i) to optimize the annotated dataset by selecting in priority notes with many mentions of the targeted conditions and ii) to select notes that contain the most interesting patterns to learn, for instance those that were not detected by the simple rule-based approach. Therefore, prior to any annotation campaign we computed two variables for each note of the training dataset:

- E the number of entities detected by the NER and Qualification modules of the simple **NLP-RB** pipeline in the case of discharge summaries and consultation reports.
- FN the number of *probable false negatives* of the **NLP-RB** pipeline, which we computed for inpatient stays only by first applying the **CLAIM** pipeline and then counting the number of conditions detected by the later but not by the **NLP-RB** pipeline. Indeed, as the **CLAIM** pipeline features a low specificity, comparing both leads to a lower bond of the number of false negatives.

In the case of outpatient stays, we used a power distribution ($p(x) = 5x^4, x \in [0, 1]$), a group was selected following the probability law:

$$\mathbb{P}(E) = \left(\frac{E+1}{E_{max}}\right)^5 - \left(\frac{E}{E_{max}}\right)^5$$

with E_{max} the maximal number of entities detected in a note of the dataset. Once the group was selected, a note was chosen among this group using a uniform sampling. This process was repeated until the number of required notes were selected.

In the case of inpatient stays, a first half of the notes were selected following the same method and a second half of the notes was selected to maximize the number of *probable false negatives*. For the selection of the latter, eligible notes were grouped by FN and a group was similarly selected following the probability law:

$$\mathbb{P}(FN) = \left(\frac{FN+1}{FN_{max}}\right)^5 - \left(\frac{FN}{FN_{max}}\right)^5$$

with FN_{max} the maximal number of *probable false negatives* detected in a note of the dataset. Adopting the same method as before, one note was then selected uniformly in the group. To consider equally both aspects we alternatively selected a single note following each method until the required amount of notes was reached.

The exponent four was chosen as it looked relevant after a visual inspection of the selected datasets. We emphasize that this obviously biased upsampling does not affect the reliability of the final performances that were computed using a uniformly sampled dataset.

3.2 De-identification and consolidation of the training dataset

During the annotation process, along with each extraction, a snippet of text is shown to the annotator. This snippet is centered around the extraction (a sentence before and a sentence after the entity). To prepare concertation meetings, which were centered around analysing and discussing incorrect extractions, the annotators were also asked to check this snippet on incorrect extractions to remove any identifying data (e.g., names, places of residence, dates of birth, etc.) and, if applicable, replacing it with a placeholder. For extra caution, if any doubt is emitted by an annotator on the potential re-identification of a patient using the remaining information contained in a snippet, the snippet was discarded.

We advocate for the capacity to centralize the annotated snippets in a development environment accessed only by data scientists/engineers in charge of the model training. Yet, this workflow is not yet validated and we can consequently exchange among environments only highly de-identified data. To be able to emulate the workflow shown in Figure 2, we therefore conducted an a-posteriori time-consuming de-identification of all snippets that relied on two stages. First, entity types listed in Table S12 were manually annotated and automatically replaced in the snippets by a plausible placeholder. Second, when the snippet could lead to indirect identification (e.g., listing exhaustively the patient's family history), it was excluded from the dataset, meaning they could not be used to train the ML-based qualification module.

Due to the iterative structure of the annotation process, some annotations carried out during the first session may not be relevant anymore when reaching the end of the process. For instance, it was decided during the process to

exclude *claudications* for the *Peripheral vascular disease* item, or to exclude *occasional* drinking from the *alcohol consumption* item. We also corrected those definition drift during the review mentioned above.

3.3 Evaluation of privacy exposure

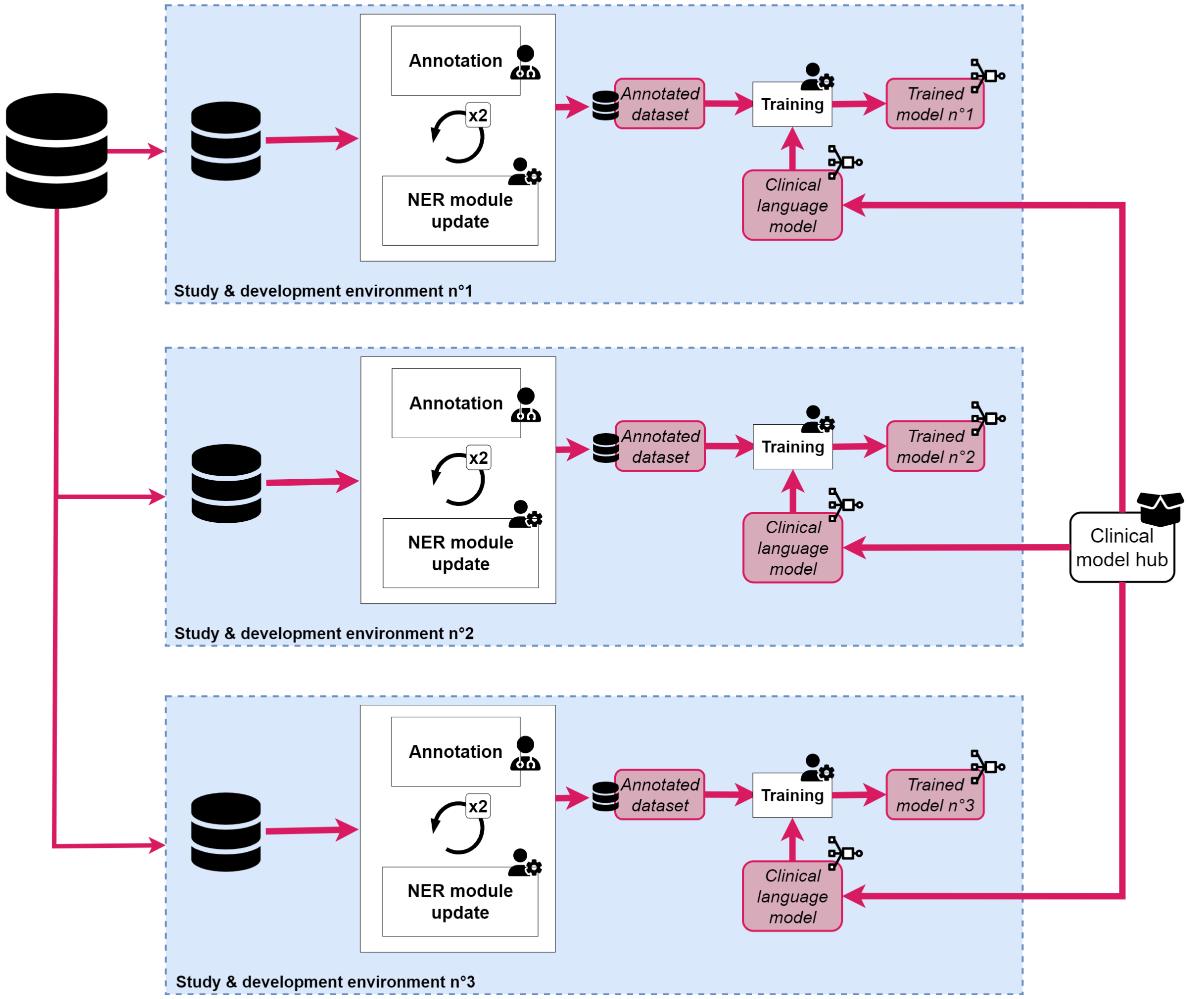

(a) **Alternative setting n°1:** No collaboration

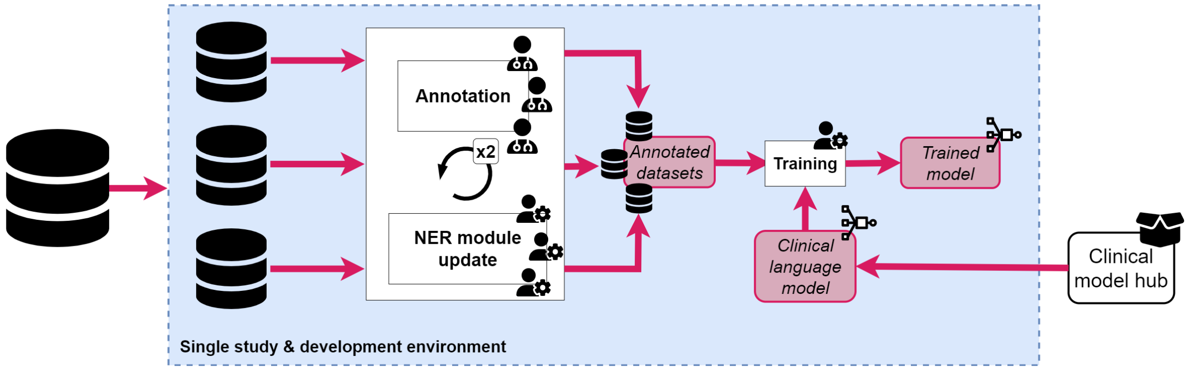

(b) **Alternative setting n°2:** Collaboration without privacy-enhancing workflow

Figure S4: Workflows of two alternative settings. **(a) Alternative non collaborative setting:** annotation, NER module update, and model training take place in each of the three isolated study environments, with dedicated clinicians and data scientists that only access a single cohort's data. **(b) Alternative collaborative setting:** Clinicians and data scientists all work in a single environment, with access to data from the three cohorts.

Person accessing data	Cardiology cohort's EHRs	Oncology cohort's EHRs	Rheumatology cohort's EHRs	Clinical Language Model	Trained pipeline (for validation and serving)
	**Privacy-sensitive data**				
Cardiology study investigators	511.412 notes	X	X	X	Unique **NLP-ML-CLINICAL** pipeline
Oncology study investigators	X	8.385.324 notes	X	X	
Rheumatology study investigators	X	X	2.058.009 notes	X	
Shared Data scientist	1457 snippets	1607 snippets	1843 snippets	One model	

(a) **Our setting:** Collaboration and privacy-enhanced workflow

Person accessing data	Cardiology cohort's EHRs	Oncology cohort's EHRs	Rheumatology cohort's EHRs	Clinical Language Model	Trained pipeline (for validation and serving)
Cardiology study investigators	511.412 notes	X	X	One model	Study-specific **NLP-ML-CLINICAL** pipeline
Oncology study investigators	X	8.385.324 notes	X	One model	Study-specific **NLP-ML-CLINICAL** pipeline
Rheumatology study investigators	X	X	2.058.009 notes	One model	Study-specific **NLP-ML-CLINICAL** pipeline

(b) **Alternative setting n°1:** No collaboration

Person accessing data	Cardiology cohort's EHRs	Oncology cohort's EHRs	Rheumatology cohort's EHRs	Clinical Language Model	Trained pipeline (for validation and serving)
Cardiology study investigators	511.412 notes	8.385.324 notes	2.058.009 notes	One model	Unique **NLP-ML-CLINICAL** pipeline
Oncology study investigators					
Rheumatology study investigators					
Shared Data scientist					

(c) **Alternative setting n°2:** Collaboration without privacy-enhancing workflow

Table S4: Privacy exposure in the case of (a) our setting and for two alternative settings described in Figure S4: (b) an non-collaborative setting and (c) a single-environment collaborative setting. For each setting, exposition to various privacy-sensitive data is reported. The red, orange and yellow colors qualitatively indicate the decreasing privacy-sensitivity of data (see text).

The workflow presented in this article limits privacy exposure by providing different technical environments dedicated to different tasks (data annotation, algorithm development, algorithm training, algorithm serving). Only the datasets and models that are necessary for each task are made available in the dedicated environment, and only those persons that need to access these items can access this environment. The transfer of data and models between environments,

along with the personal accesses granted to each environment, are controlled by the core team in charge of the platform operation and by the institutional review board (IRB).

Our main setting enhances privacy protection compared to an alternative setting with a similar level of collaboration but without distinct environments, and makes privacy exposure similar to a non-collaborative setting while still providing the performance enhancement of collaboration ((Figure S4 and (Table S4). Indeed, we limit the number of persons that access each clinical note. These data are highly sensitive as, even if they are pseudonymized, it is often possible for a malicious clinician or data scientist to re-identify someone using knowledge such as the patient's weight, height, medical history, family history, dates of hospitalization, etc.[5] Such a re-identification may lead the attacker to learn sensitive medical information about the re-identified patient.

In our workflow, clinicians and data scientists may access three kinds of data containing personally identifying information (PII): clinical notes, but also annotated snippets of text (i.e., some words/sentences extracted from a note) and trained models. Snippets of texts are much less privacy-sensitive than notes as they usually do not contain enough information to re-identify a patient. Trained models have long been considered as privacy-insensitive but recent results suggest that this is no more the case as large language models may store PII within their trained parameters.[6,7] A malicious clinician or data scientist that accesses such a model may retrieve PII using specific attacks. Although these attacks are still difficult to conduct, they may become easier in the future and the risk may also increase with the size of language models as larger models may store more information.[8]

To limit privacy exposure, it therefore appears relevant to limit the number of persons that access each privacy-sensitive item. Table S4 shows, for the main setting and the alternative settings presented in the article, the persons that access each dataset/model.

3.4 Development environment

In this particular study, for the sake of simplicity we used the rheumatology environment as a development environment. Since it was already granted access to the clinical language model, and since the annotated datasets underwent a time-consuming de-identification to enable their transfer between environments (see above), we could faithfully emulate the proposed workflow presented in Figure 2 while complying to the current rules of the CDW. More generally, we can advocate for the use of such a development environment for future training of machine learning models, together with the trained model being made available through a secure model hub. Access to this environment could be granted only to a limited number of machine learning engineers to limit privacy exposure.

4 Appendix D: Computation of the CCI

Beyond the analysis of each condition, we moreover compared three approaches to compute the CCI: i) using *Quan et al.* formula to aggregate the conditions detected by the **NLP-ML-CLINICAL** pipeline (CCI_{ML}), ii) using the same formula to aggregate the conditions detected by the **CLAIM** pipeline (CCI_{Claim}) or iii) using direct detection of the CCI score computed by clinicians at the point of care and reported in the clinical notes ($CCI_{Reported}$).[9] For this evaluation we considered an additional sub-cohort composed of the three cohorts' inpatient stays with an admission in an intensive care unit (ICU) department and for which a simple rule-based NLP algorithm detected a CCI reported by the clinicians. The coherence between these scores was assessed by computing $\Delta(CCI)$, the differences of CCI_{ML} with CCI_{Claim} and $CCI_{Reported}$, respectively. Additionally, correlation between CCI computed by two distinct methods were assessed via Spearman correlation coefficient.

The ICU cohort consisted of 46, 1288 and 279 stays for the cardiology, oncology and rheumatology studies respectively. Figure S5 shows the distribution of $\Delta(CCI)$ comparing CCI_{ML} with either $CCI_{Reported}$ or CCI_{Claim}. Overall, the distributions have a median (and interquartile range) of 1 (4) and 0 (2), respectively. Specifically, CCI_{ML} and CCI_{Claim} fully agree for 33% of the stays versus 16% for CCI_{ML} and $CCI_{Reported}$. When disagreeing, CCI_{ML} is higher than CCI_{Claim} for 70% of the stays. More generally, CCI computed via two distinct methods show poor correlation, with correlation coefficient of 0.22, 0.41 and 0.38 for CCI_{ML} vs $CCI_{Reported}$, CCI_{ML} vs CCI_{Claim} and CCI_{Claim} vs $CCI_{Reported}$, respectively.

The agreement with CCI_{ML} is still higher for CCI_{Claim} than for $CCI_{Reported}$, having both a smaller interquartile range and a median closer to zero. Most stays featuring a disagreement between CCI_{ML} and CCI_{Claim} have a higher CCI_{ML}. This discrepancy could have partially occurred because we added some conditions initially absent to the CCI, such as arterial hypertension in peripheral vascular disease or atrial fibrillation in congestive heart failure. Those conditions are known to be prone to under-coding, thus biasing CCI_{Claim} towards lower values without impacting CCI_{ML} as much. More generally, pairwise correlation is poor regardless of method, with each method exhibiting limitations: CCI_{ML} was computed on patients specifically visiting ICU departments while being built and trained

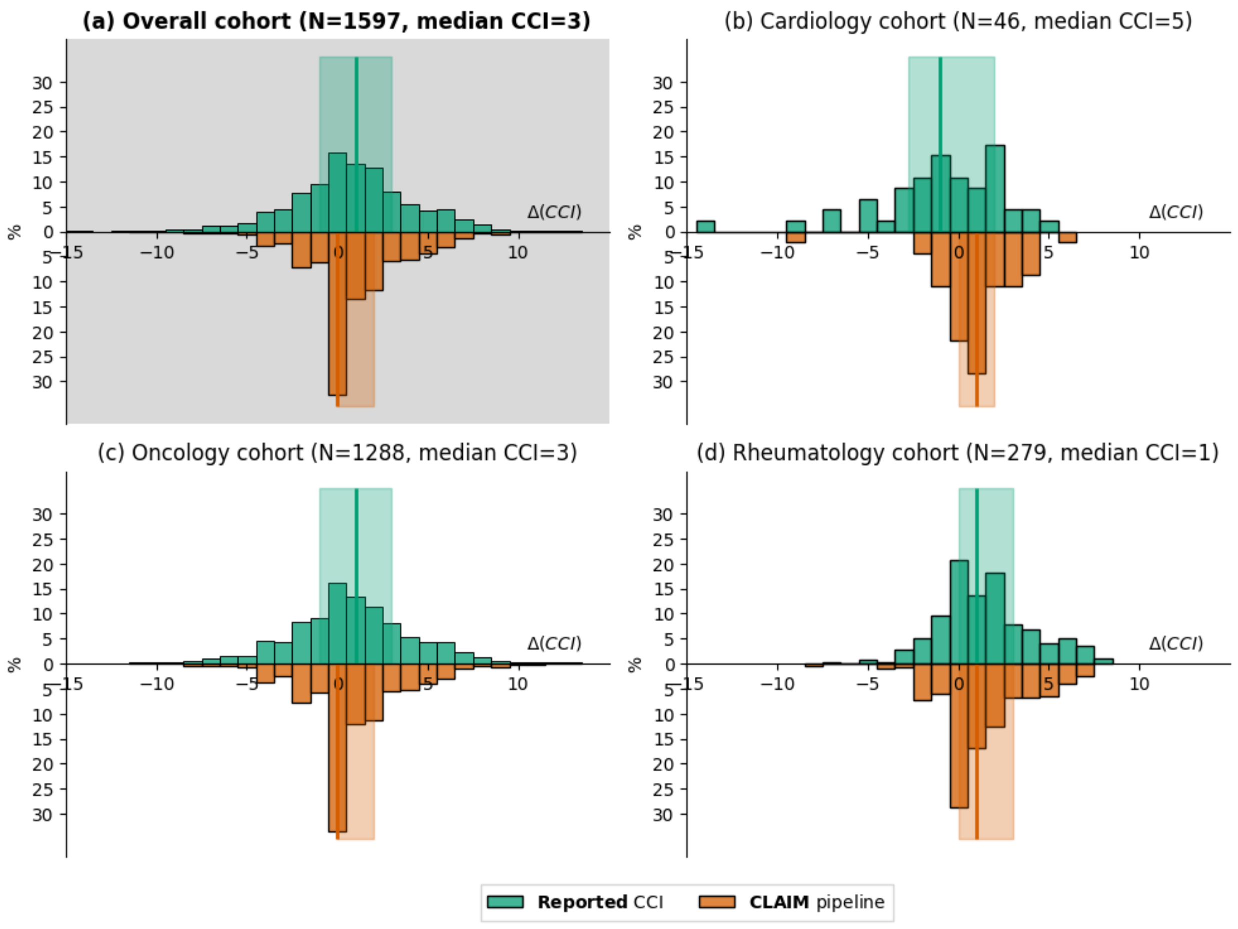

Figure S5: Difference between the Charlson Comorbidity Index obtained using the main **NLP-ML-CLINICAL** pipeline and its value computed either by the clinician at the point of care (green, number of inpatient stays and median score in brackets) or a posteriori from the alternative **CLAIM** pipeline (orange). Distributions, medians and interquartile ranges are indicated by histograms, vertical lines and shaded areas, respectively. The cardiology (b), oncology (c), rheumatology (d) or overall (a) cohorts are considered separately.

on a more general corpus, which could impair performances. As mentioned above, CCI_{Claim} suffers from coding biases. $CCI_{Reported}$ was computed while patients were in ICU, in an emergency situation where information about each condition was not necessarily available at the time of calculation. Additionally, we lacked information regarding the method and clinical rationale used by the many clinicians who computed these $CCI_{Reported}$. In the end, no method seems to guarantee a ground truth regarding the CCI, prompting one to be cautious about the use of those calculated scores.

5 Appendix E: Additional results

	Cardiology cohort		Oncology cohort		Rheumatology cohort		Overall cohort	
**Stay type**	**inpatient**	**outpatient**	**inpatient**	**outpatient**	**inpatient**	**outpatient**	**inpatient**	**outpatient**
**Age at admission**	84.6 (7.3)	80.9 (6.9)	61.4 (18.7)	61.9 (15.2)	57.5 (19.4)	53.4 (19.2)	67.8 (20.0)	65.4 (18.6)
**Gender distribution (M-F)**	48 - 52	48 - 52	60 - 40	46 - 54	50 - 50	36 - 64	52 - 47	43 - 56
**Total annotated records**	50	50	50	50	50	50	150	150
**Myocardial infarction**	15 (3.5)	10 (2.1)	10 (2.7)	3 (1.3)	10 (2.7)	4 (1.5)	35 (3.1)	17 (1.8)
**Congestive heart failure**	41 (6.3)	22 (3.0)	15 (4.1)	1 (4.0)	32 (3.3)	2 (1.0)	88 (4.8)	25 (2.8)
**Peripheral vascular disease**	41 (2.3)	17 (2.0)	29 (2.6)	7 (1.4)	37 (2.9)	12 (2.7)	107 (2.6)	36 (2.1)
**Cerebrovascular disease**	12 (1.8)	4 (1.8)	5 (2.4)	4 (1.2)	16 (5.7)	5 (1.6)	33 (3.8)	13 (1.5)
**Dementia**	15 (1.7)	2 (2.5)	4 (1.8)	0	9 (3.1)	3 (1.3)	28 (2.2)	5 (1.8)
**Chronic pulmonary disease**	22 (4.2)	15 (1.7)	6 (3.5)	6 (1.5)	16 (3.4)	11 (1.5)	44 (3.8)	32 (1.6)
**Rheumatologic disease**	1 (1.0)	3 (1.3)	3 (2.7)	2 (1.5)	19 (4.2)	15 (2.1)	23 (3.9)	20 (2.0)
**Peptic ulcer disease**	7 (1.4)	0	3 (1.7)	1 (1.0)	3 (1.3)	0	13 (1.5)	1 (1.0)
**Liver disease**	0	1 (3.0)	2 (8.0)	1 (2.0)	8 (6.5)	3 (2.3)	10 (6.8)	5 (2.4)
**Diabetes**	16 (2.2)	13 (1.4)	14 (2.5)	4 (1.8)	15 (3.0)	8 (1.5)	45 (2.6)	25 (1.5)
**Hemiplegia***	1 (2.0)	1 (2.0)	1 + *10* (6.0)	0 + *14* (-)	2 + *15* (1.0)	0 + *15* (-)	4 + *25* (2.5)	1 + *29* (2.0)
**Renal disease**	10 (1.8)	4 (2.0)	5 (1.2)	2 (2.0)	1 (4.0)	0	16 (1.8)	6 (2.0)
**Solid tumor**	11 (2.6)	6 (2.2)	27 (3.4)	23 (2.6)	11 (1.5)	10 (1.4)	49 (2.8)	39 (2.2)
**Leukemia**	2 (3.0)	1 (4.0)	3 (1.3)	3 (2.7)	4 (3.5)	3 (1.7)	9 (2.7)	7 (2.4)
**Lymphoma**	7 (4.6)	8 (1.4)	5 (4.6)	5 (2.0)	7 (1.9)	1 (1.0)	19 (3.6)	14 (1.6)
**AIDS***	1 (1.0)	0	1 + *8* (2.0)	0 + *8* (-)	0 + *13* (-)	0 + *9* (-)	2 + *21* (1.5)	0 + *17* (-)
**Alcohol Consumption**	14 (1.1)	1 (1.0)	13 (1.6)	1 (2.0)	18 (1.9)	3 (1.0)	45 (1.6)	5 (1.2)
**Tobacco consumption**	24 (1.5)	8 (1.4)	20 (1.6)	11 (1.1)	24 (1.6)	6 (1.3)	68 (1.5)	25 (1.2)
**Total**	49 (14.9)	41 (5.6)	46 (9.6)	38 (3.7)	48 (14.9)	40 (3.8)	143 (13.2)	119 (4.4)

* For those conditions, the number of upsampled notes is also shown *in italics*

Table S5: Composition of the validation dataset sampled in the three disease-specific cohorts without considering qualification variables (i.e., including negated and hypothetical entities, or entities not related to the patient). For each condition, the number of clinical notes with at least one validated entity is shown along with, for those notes, the mean number of validated entities per note (in brackets). For instance, in the cardiology cohort, 15 discharge summaries mention a myocardial infarction and on average 3.5 validated entities are present within each one of them.

		F1	Positive predictive value	Sensitivity
**Myocardial infarction**		92,3	95,3	89,5
**Congestive heart failure**		92,3	91,9	92,6
**Peripheral vascular disease**		89,3	91,6	87,1
**Cerebrovascular disease**		87	87,9	86
**Dementia**		90,4	92,9	88,1
**Chronic pulmonary disease**		90,4	87,9	93
**Rheumatologic disease**		84,7	94,7	76,6
**Peptic ulcer disease**		100	100	100
**Liver**	*Mild*	94,1	100	88,9
	Moderate to severe	88	84,6	91,7
	Any	89,9	89,1	90,7
**Diabetes**	*Without complications*	89	94,2	84,4
	With complications	83,3	87	80
	Any	92,2	97,2	87,6
**Hemiplegia**⋆		-	91,1	-
**Renal disease**		80,6	82,9	78,4
**Solid tumor**	*Localized*	91,1	93,9	88,5
	Metastatic	90,1	91,4	88,9
	Any	90,9	93,3	88,6
**Leukemia**		94,1	97	91,4
**Lymphoma**		91,4	91,4	91,4
**AIDS**⋆		-	57,5	-
**Alcohol consumption**	*Present*	84,7	78,1	92,6
	Stopped	63,2	100	46,2
	Any	94,9	97,4	92,5
**Tobacco consumption**	*Present*	87,6	90,7	84,8
	Stopped	94,9	94,9	94,9
	Any	94,6	96,3	92,9
**Total**	**Micro average**	90,6	92,3	89
	**Macro average**	90,9	92,9	89,2
	**Weighted average**	90,7	92,4	89,3

⋆ For those conditions, only the positive predictive value was computed.

Table S6: Entity-level performances (F1-score, positive predictive value and sensitivity) of the main **NLP-ML-CLINICAL** pipeline used to predict 18 conditions from clinical notes. We considered jointly inpatient and outpatient of the three disease-specific cohorts.

		Cardiology cohort				Oncology cohort				Rheumatology cohort			
		**F1**	**PPV**	**Sensitivity**	**Specificity**	**F1**	**PPV**	**Sensitivity**	**Specificity**	**F1**	**PPV**	**Sensitivity**	**Specificity**
**Myocardial infarction**		95,2	95,2	95,2	98,7	85,7	81,8	90	97,8	94,7	100	90	100
**Congestive heart failure**		95,4	96,3	94,5	95,6	100	100	100	100	96,8	93,8	100	98,8
**Peripheral vascular disease**		97,2	96,4	98,1	95,7	98,5	97	100	98,5	97,3	100	94,7	100
**Cerebrovascular disease**		100	100	100	100	90,9	83,3	100	98,9	90,3	87,5	93,3	97,6
**Dementia**		93,3	93,3	93,3	98,8	85,7	75	100	99	96	92,3	100	98,9
**Chronic pulmonary disease**		95,4	93,9	96,9	97,1	84,2	80	88,9	97,8	97,1	94,4	100	98,8
**Rheumatologic disease**		66,7	100	50	100	66,7	100	50	100	98	96,2	100	98,7
**Peptic ulcer disease**		100	100	100	100	100	100	100	100	100	100	100	100
**Liver**	*Mild*	-	-	-	-	100	100	100	100	100	100	100	100
	Moderate to severe	100	100	100	100	100	100	100	100	100	100	100	100
	Any	100	100	100	100	100	100	100	100	100	100	100	100
**Diabetes**	*Without complications*	97,6	100	95,2	100	100	100	100	100	90,9	100	83,3	100
	With complications	100	100	100	100	100	100	100	100	75	60	100	97,9
	Any	98,2	100	96,4	100	100	100	100	100	100	100	100	100
**Hemiplegia**⋆		-	-	-	-	-	79,3	-	-	-	100	-	-
**Renal disease**		87	100	76,9	100	80	66,7	100	96,8	100	100	100	100
**Solid tumor**	*Localized*	94,7	100	90	100	92,9	92,9	92,9	97,2	92,3	85,7	100	97,7
	Metastatic	100	100	100	100	96,3	100	92,9	100	100	100	100	100
	Any	96	100	92,3	100	96,4	97,6	95,2	98,3	92,9	86,7	100	97,7
**Leukemia**		80	66,7	100	99	100	100	100	100	100	100	100	100
**Lymphoma**		100	100	100	100	100	100	100	100	85,7	100	75	100
**AIDS**⋆		-	-	-	-	-	53,3	-	-	-	69,6	-	-
**Alcohol consumption**	*Present*	100	100	100	100	75	60	100	97,9	85,7	75	100	99
	Stopped	100	100	100	100	50	100	33,3	100	85,7	100	75	100
	Any	100	100	100	100	100	100	100	100	100	100	100	100
**Tobacco consumption**	*Present*	83,3	83,3	83,3	98,9	71,4	62,5	83,3	96,8	80	85,7	75	98,9
	Stopped	100	100	100	100	95,2	100	90,9	100	85,7	81,8	90	97,8
	Any	94,4	94,4	94,4	98,8	91,4	88,9	94,1	97,6	100	100	100	100
**Total**	**Micro average**	95,6	96,5	94,8	99,2	94,6	93	96,1	99,1	96,9	96,2	97,6	99,4
	**Macro average**	93,7	96	93	99	92,5	91,9	94,9	99	96,8	96,9	97,1	99,4
	**Weighted average**	95,5	96,7	94,8	97,8	94,6	93,9	96,1	98,7	96,9	96,4	97,6	99,3

⋆ For those conditions, only the positive predictive value was computed.

Table S9: Stay-level performance of the main **NLP-ML-CLINICAL** stratified by cohort.

		F1			Positive predictive value			Sensitivity		
		NLP-ML-CLINICAL	NLP-ML-GENERIC	NLP-RB	NLP-ML-CLINICAL	NLP-ML-GENERIC	NLP-RB	NLP-ML-CLINICAL	NLP-ML-GENERIC	NLP-RB
**Myocardial infarction**		**96,9**	96,5	95,7	**98,2**	96,5	94,1	95,6	96,5	**97,4**
**Congestive heart failure**		**97,5**	97	95,9	96,2	96,4	**96,5**	**98,9**	97,6	95,3
**Peripheral vascular disease**		**95,8**	95	92,8	**97,6**	96,7	97	**94,1**	93,3	89
**Cerebrovascular disease**		93,8	93,6	**94,6**	90,9	92,6	**95,6**	**96,8**	94,6	93,5
**Dementia**		**96,7**	92,6	88,9	**93,7**	90,3	89,7	**100**	94,9	88,1
**Chronic pulmonary disease**		95,2	**96,5**	94,6	91,9	**96**	93,2	**98,8**	97,1	95,9
**Rheumatologic disease**		**95,2**	92,8	93,8	95,7	**96,6**	91	94,7	89,4	**96,8**
**Peptic ulcer disease**		**100**	**100**	90,9	**100**	**100**	88,2	**100**	**100**	93,8
**Liver**	*Mild*	**97,1**	**97,1**	91,4	**100**	**100**	94,1	**94,4**	**94,4**	88,9
	Moderate to severe	**90,9**	89,2	84,6	85,4	**86,8**	78,6	**97,2**	91,7	91,7
	Any	**92,9**	91,7	86,7	89,7	**90,9**	83,1	**96,3**	92,6	90,7
**Diabetes**	*Without complications*	**92,3**	90,5	86,9	**97,7**	97,6	87,4	**87,5**	84,4	86,5
	With complications	**96,2**	90,9	85,2	**92,6**	83,3	79,3	**100**	**100**	[illegible]
	Any	**93,2**	90,6	86,5	**96,5**	93,8	85,5	**90,1**	87,6	[illegible]
**Hemiplegia***		-	-	-	**97,4**	93,6	94,9	-	-	
**Renal disease**		**100**	98,7	95,9	**100**	97,4	97,2	**100**	**100**	94,6
**Solid tumor**	*Localized*	**95,6**	94	92,1	**93,7**	91,5	89,2	**97,5**	96,7	95,1
	Metastatic	90,9	**93,3**	92,3	85,4	**89,7**	85,7	97,2	97,2	**100**
	Any	**94,5**	93,9	92,1	**91,7**	91,1	88,4	**97,5**	96,8	96,2
**Leukemia**		97,1	**100**	98,6	**100**	**100**	**100**	94,3	**100**	97,1
**Lymphoma**		93,1	**93,4**	84	**93,1**	89,1	75,3	93,1	**98,3**	94,8
**AIDS***		-	-	-	94	**95,7**	92,5	-	-	-
**Alcohol consumption**	*Present*	**94,3**	86,3	83,6	**96,2**	91,7	82,1	**92,6**	81,5	85,2
	Stopped	**100**	96	91,7	**100**	**100**	**100**	**100**	92,3	84,6
	Any	**96,2**	89,5	86,1	**97,4**	94,4	87,2	**95**	85	85
**Tobacco consumption**	*Present*	**92,3**	85,4	76,4	**93,3**	88,4	65,6	**91,3**	82,6	**91,3**
	Stopped	**98,7**	**98,7**	94,6	**100**	**100**	**100**	**97,4**	**97,4**	89,7
	Any	**95,2**	91,6	83,7	**96,4**	93,8	77,8	**94,1**	89,4	90,6
**Total**	**Micro average**	**95,7**	94,8	92,4	**95,1**	94,9	91,5	**96,3**	94,7	93,3
	**Macro average**	**95,8**	94,6	91,3	**95,5**	94,7	90	**96,2**	94,6	92,9
	**Weighted average**	**95,7**	94,8	92,5	**95,2**	94,9	91,9	**96,3**	94,7	93,3

* For those conditions, only the positive predictive value was computed.

Table S7: Stay-level performance of the qualification algorithms only of the main **NLP-ML-CLINICAL** pipeline, the **NLP-ML-GENERIC** pipeline and the **NLP-RB** pipeline: algorithms ran on validated entities only.

Stay type		F1 inpatient	F1 outpatient	Positive predictive value inpatient	Positive predictive value outpatient	Sensitivity inpatient	Sensitivity outpatient	Specificity inpatient	Specificity outpatient
**Myocardial infarction**		94.9 (89.2 - 98.4)	87.0 (76.0 - 96.0)	90.3 (80.6 - 96.8)	100.0 (-)	100.0 (-)	76.9 (61.3 - 92.3)	97.5 (95.2 - 99.2)	100.0 (-)
**Congestive heart failure**		95.7 (91.1 - 98.3)	97.9 (95.6 - 98.0)	94.9 (89.7 - 98.4)	100.0 (-)	96.6 (91.0 - 98.3)	95.8 (91.6 - 96.0)	96.7 (93.7 - 98.9)	100.0 (-)
**Peripheral vascular disease**		97.2 (95.4 - 98.4)	98.6 (97.1 - 98.6)	97.8 (95.6 - 98.9)	97.1 (94.3 - 97.2)	96.7 (93.3 - 98.9)	100.0 (-)	96.7 (93.5 - 98.3)	99.1 (98.3 - 99.1)
**Cerebrovascular disease**		96.0 (91.6 - 98.0)	88.9 (75.0 - 90.9)	92.3 (84.5 - 96.2)	88.9 (70.0 - 90.9)	100.0 (-)	88.9 (66.7 - 90.9)	98.4 (96.9 - 99.2)	99.3 (97.9 - 99.3)
**Dementia**		92.3 (85.7 - 94.5)	100.0 (-)	88.9 (78.6 - 92.9)	100.0 (-)	96.0 (88.4 - 96.6)	100.0 (-)	97.6 (95.3 - 98.4)	100.0 (-)
**Chronic pulmonary disease**		92.8 (86.2 - 97.3)	96.0 (89.8 - 96.3)	88.9 (80.5 - 97.3)	96.0 (87.4 - 96.3)	97.0 (90.9 - 97.3)	96.0 (91.2 - 96.3)	96.6 (94.2 - 99.1)	99.2 (97.6 - 99.2)
**Rheumatologic disease**		87.5 (71.4 - 94.4)	96.8 (89.7 - 97.0)	93.3 (73.3 - 94.5)	100.0 (-)	82.4 (62.4 - 94.4)	93.8 (81.2 - 94.1)	99.2 (97.1 - 99.2)	100.0 (-)
**Peptic ulcer disease**		100.0 (-)	100.0 (-)	100.0 (-)	100.0 (-)	100.0 (-)	100.0 (-)	100.0 (-)	100.0 (-)
**Liver**	*Mild*	100.0 (-)	100.0 (-)	100.0 (-)	100.0 (-)	100.0 (-)	100.0 (-)	100.0 (-)	100.0 (-)
	Moderate to severe	100.0 (-)	100.0 (-)	100.0 (-)	100.0 (-)	100.0 (-)	100.0 (-)	100.0 (-)	100.0 (-)
	Any	100.0 (-)	100.0 (-)	100.0 (-)	100.0 (-)	100.0 (-)	100.0 (-)	100.0 (-)	100.0 (-)
**Diabetes**	*Without complications*	96.4 (92.5 - 98.2)	96.8 (89.5 - 97.0)	100.0 (-)	100.0 (-)	93.1 (86.1 - 96.6)	93.8 (80.9 - 94.1)	100.0 (-)	100.0 (-)
	With complications	94.1 (87.3 - 94.7)	92.3 (83.1 - 93.3)	88.9 (77.5 - 90.0)	85.7 (71.1 - 87.5)	100.0 (-)	100.0 (-)	99.3 (98.6 - 99.3)	99.3 (98.6 - 99.3)
	Any	98.6 (95.7 - 98.7)	100.0 (-)	100.0 (-)	100.0 (-)	97.3 (91.8 - 97.4)	100.0 (-)	100.0 (-)	100.0 (-)
**Hemiplegia***		-	-	82.8 (71.4 - 93.4)	96.3 (88.8 - 96.4)	-	-	-	-
**Renal disease**		85.7 (72.0 - 93.8)	83.3 (44.4 - 92.3)	92.3 (81.7 - 93.8)	71.4 (28.6 - 85.7)	80.0 (60.0 - 93.8)	100.0 (-)	99.3 (98.5 - 99.3)	98.6 (96.6 - 99.3)
**Solid tumor**	*Localized*	96.0 (89.4 - 98.0)	90.2 (83.2 - 94.7)	92.3 (80.8 - 96.2)	92.0 (80.7 - 96.2)	100.0 (-)	88.5 (80.0 - 96.4)	98.4 (96.1 - 99.2)	98.4 (96.1 - 99.2)
	Metastatic	100.0 (-)	92.3 (72.0 - 93.3)	100.0 (-)	100.0 (-)	100.0 (-)	85.7 (56.4 - 87.5)	100.0 (-)	100.0 (-)
	Any	97.2 (94.2 - 98.6)	93.8 (86.6 - 97.1)	94.6 (89.1 - 97.3)	96.8 (89.9 - 97.1)	100.0 (-)	90.9 (81.2 - 97.1)	98.3 (96.6 - 99.1)	99.1 (97.5 - 99.1)
**Leukemia**		94.1 (71.4 - 94.7)	100.0 (-)	88.9 (55.6 - 90.0)	100.0 (-)	100.0 (-)	100.0 (-)	99.3 (97.2 - 99.3)	100.0 (-)
**Lymphoma**		95.7 (85.7 - 96.0)	100.0 (-)	100.0 (-)	100.0 (-)	91.7 (75.0 - 92.3)	100.0 (-)	100.0 (-)	100.0 (-)
**AIDS**		-	-	55.6 (44.2 - 70.4)	65.4 (49.9 - 80.8)	-	-	-	-
**Alcohol consumption**	*Present*	76.9 (40.0 - 93.3)	100.0 (-)	62.5 (25.0 - 87.5)	100.0 (-)	100.0 (-)	100.0 (-)	97.9 (95.9 - 99.3)	100.0 (-)
	Stopped	80.0 (50.0 - 94.1)	-	100.0 (-)	-	66.7 (33.3 - 88.9)	-	100.0 (-)	-
	Any	100.0 (-)	100.0 (-)	100.0 (-)	100.0 (-)	100.0 (-)	100.0 (-)	100.0 (-)	100.0 (-)
**Tobacco consumption**	*Present*	66.7 (44.2 - 88.1)	90.0 (70.4 - 95.2)	70.0 (49.8 - 90.9)	81.8 (54.3 - 90.9)	63.6 (36.1 - 91.7)	100.0 (-)	97.8 (96.4 - 99.3)	98.6 (96.5 - 99.3)
	Stopped	93.9 (84.3 - 96.2)	94.1 (87.3 - 94.7)	92.0 (75.9 - 96.2)	100.0 (-)	95.8 (89.4 - 96.2)	88.9 (77.5 - 90.0)	98.4 (95.4 - 99.2)	100.0 (-)
	Any	94.3 (89.5 - 95.8)	97.3 (88.2 - 97.4)	94.3 (88.8 - 97.1)	94.7 (78.9 - 95.0)	94.3 (85.6 - 97.1)	100.0 (-)	98.3 (96.6 - 99.1)	99.2 (97.0 - 99.2)
**Total**	**Micro average**	95.5 (93.8 - 95.9)	96.2 (93.9 - 96.9)	94.8 (92.8 - 95.6)	96.9 (93.7 - 97.4)	96.2 (94.2 - 96.9)	95.6 (93.0 - 96.6)	98.8 (98.3 - 99.0)	99.7 (99.4 - 99.7)
	**Macro average**	95.1 (92.7 - 95.7)	96.2 (93.0 - 97.0)	94.8 (92.2 - 95.3)	96.6 (93.2 - 97.5)	95.7 (93.2 - 96.4)	96.4 (94.2 - 97.3)	98.6 (98.1 - 98.9)	99.7 (99.3 - 99.7)
	**Weighted average**	95.5 (93.7 - 95.9)	96.2 (94.0 - 96.9)	94.9 (93.1 - 95.7)	97.2 (94.9 - 97.5)	96.2 (94.2 - 96.9)	95.6 (93.0 - 96.6)	98.0 (97.2 - 98.5)	99.5 (99.2 - 99.5)

In case of metrics at 100, no bootstraping was performed.

* For those conditions, only the positive predictive value was computed.

Table S8: Stay-level performances (F1-score, positive predictive value, sensitivity and specificity) of the main **NLP-ML-CLINICAL** pipeline used to predict 18 conditions from clinical notes stratified by stay type. 95% confidence intervals were estimated by bootstrapping.

	Cardiology cohort	Oncology cohort	Rheumatology cohort	Overall
**Myocardial infarction**	70	108	83	261
**Congestive heart failure**	308	238	177	723
**Peripheral vascular disease**	223	199	409	831
**Cerebrovascular disease**	87	118	163	368
**Dementia**	36	63	33	132
**Chronic pulmonary disease**	164	110	140	414
**Rheumatologic disease**	33	15	161	209
**Peptic ulcer disease**	6	21	11	38
**Liver disease**	24	30	62	116
**Diabetes**	126	140	132	398
**Hemiplegia**	9	2	7	18
**Renal disease**	91	260	144	495
**Solid tumor**	84	111	63	258
**Leukemia**	30	34	41	105
**Lymphoma**	48	53	84	185
**AIDS**	0	1	3	4
**Alcohol Consumption**	39	36	61	136
**Tobacco consumption**	79	68	69	216
**Total**	1457	1607	1843	4907

Table S10: Number of annotated entities present in the training dataset, for each cohort and each condition.

6 Appendix F: Annotation guideline

6.1 Annotation content

Each entity should be annotated along with, when relevant, its severity, the start and end of the entity in the text, and qualifiers deduced from its context (negation, family, hypothesis). Below are a few examples with their corresponding annotation in Table S11.

Examples:

- M. DUPONT est **diabétique**. (*Mr DUPONT is **diabetic**.*)
- Probable **SAPL** chez le père du patient. (*Probable **APS** in the patient's father.*)
- A été traité à **AVC**[1] en 2020. (*Was treated at **AVC** hospital in 2020*)

[1] AVC is an acronym for stroke, but also the acronym of one of AP-HP's hospitals.

	Label	Status	Negation	Family	Hypothesis	Start	End	Anonymized Text
**Definition:**	The associated comorbidity	The status of the comorbidity	Is the entity negated ?	Is the entity referring to someone different that the patient	Is the entity uncertain ?	Start position of the entity	End position of the entity	Snippet with identifying traits replaced
**Example 1**	Diabetes	Without complications	NO	NO	NO	15	25	M. MARTIN est diabétique
**Example 2**	Peripheral vascular disease	Present	NO	YES	YES	9	13	Probable SAPL chez le père du patient
**Example 3**	Cerebrovascular disease	Absent	NO	NO	NO	15	18	A été traité à AVC en 2010

Table S11: Details of the annotation of a few examples.

6.2 General considerations

Acute manifestations of diseases are not considered:

- Le patient est passé aux urgences suite à une insuffisance rénale aigüe (*The patient was admitted to emergency with acute renal failure.*) → **NO**
- Le patient a un insuffisance rénale de stade 4 (*Patient has stage 4 renal failure*) → **YES**

Are not labeled terms specific to the semiology of radiology and medical imaging, nor the terms present in the physical examination. Indeed, the scope of the project is only to focus on the certain diagnostic already made and explicitly mentioned in the text, not to predict one:

- IRM cérébrale: présence d'une petite lacune lenticulaire (*Brain MRI: presence of a small lenticular lacuna*) → **Not labeled as cerebrovascular disease.**

Annotation should be performed at the entity level, i.e. by considering its local context only:

For instance, let us consider the following snippet:

- Le patient est suivi pour un diabète (1) de type II. Il a également [...] et une rétinopathie diabétique (2). (*The patient is being followed for type II diabetes (1). He also has [...] and diabetic retinopathy (2)*)

In this case, the status of extraction (1) should be set to *without complications* (nothing, in the immediate vicinity of the entity, mentions a complication), while the status of extraction (2) should be *with complications*.

In the case of comorbidities with non-binary status, if the status isn't explicitly mentioned, it should be set to its default value:

- Le patient est atteint d'un cancer du poumon (*Patient has lung cancer*) → ***Solid tumor* set to *localized***

Label	Description
ADRESSE	Street address, e.g., 33 boulevard de Picpus
DATE	Any absolute date other than a birthdate
DATE_NAISSANCE	Birthdate
HOPITAL	Hospital name, e.g., Hôpital Rothschild
IPP	Internal AP-HP identifier for patients, displayed as a number
MAIL	Email address
NDA	Internal AP-HP identifier for visits, displayed as a number
NOM	Any last name (patients, doctors, third parties)
PRENOM	Any first name (patients, doctors, etc)
SECU	Social security number
TEL	Any phone number
VILLE	Any city
ZIP	Any zip code

Table S12: Types of identifying entities replaced by the de-identification process

REFERENCES

[1] Emmanuelle Kempf, Guillaume Lamé, Richard Layese, et al. New cancer cases at the time of sars-cov2 pandemic and related public health policies: a persistent and concerning decrease long after the end of the national lockdown. *European Journal of Cancer*, 150:260–267, 2021.

[2] Romain Bey, Romain Goussault, François Grolleau, et al. Fold-stratified cross-validation for unbiased and privacy-preserving federated learning. *Journal of the American Medical Informatics Association*, 27(8):1244–1251, 2020.

[3] Wendy W Chapman, Will Bridewell, Paul Hanbury, et al. A simple algorithm for identifying negated findings and diseases in discharge summaries. *Journal of biomedical informatics*, 34(5):301–310, 2001.

[4] Basile Dura, Perceval Wajsburt, Thomas Petit-Jean, et al. EDS-NLP: efficient information extraction from French clinical notes, July 2023.

[5] Xavier Tannier, Perceval Wajsbürt, Alice Calliger, et al. Development and validation of a natural language processing algorithm to pseudonymize documents in the context of a clinical data warehouse. *arXiv preprint arXiv:2303.13451*, 2023.

[6] Nicholas Carlini, Florian Tramer, Eric Wallace, et al. Extracting training data from large language models. In *30th USENIX Security Symposium (USENIX Security 21)*, pages 2633–2650, 2021.

[7] Michael Moor, Oishi Banerjee, Zahra Shakeri Hossein Abad, et al. Foundation models for generalist medical artificial intelligence. *Nature*, 616(7956):259–265, 2023.

[8] Nils Lukas, Ahmed Salem, Robert Sim, et al. Analyzing leakage of personally identifiable information in language models. *arXiv preprint arXiv:2302.00539*, 2023.

[9] Hude Quan, Bing Li, Chantal M Couris, et al. Updating and validating the charlson comorbidity index and score for risk adjustment in hospital discharge abstracts using data from 6 countries. *American journal of epidemiology*, 173(6):676–682, 2011.
