# Supplementary material for: Collaborative and privacy-enhancing workflows on a clinical data warehouse: an example developing natural language processing pipelines to detect medical conditions
Source: J Am Med Inform Assoc. 2024 Apr 4;31(6):1280–90. doi: 10.1093/jamia/ocae069 (PMC11105139; doi:10.1093/jamia/ocae069)
Supplement: ocae069_Supplementary_Data [file ocae069_supplementary_data.zip › ocae069_Supplementary_Data/TF-JAMI240070_Log_Rpt.html]

|  |  |  |  |  |
| --- | --- | --- | --- | --- |
| **File Name:** |  |  | **Status:** Success | **Processed Time:** 13-Mar-24 1:35:56 PM |

---

##### Login Report

| Error Description |
| --- |
| No Error - File moved successfully |
