# Supplementary figures and images for: Collaborative and privacy-enhancing workflows on a clinical data warehouse: an example developing natural language processing pipelines to detect medical conditions
Source: J Am Med Inform Assoc. 2024 Apr 4;31(6):1280–90. doi: 10.1093/jamia/ocae069 (PMC11105139; doi:10.1093/jamia/ocae069)

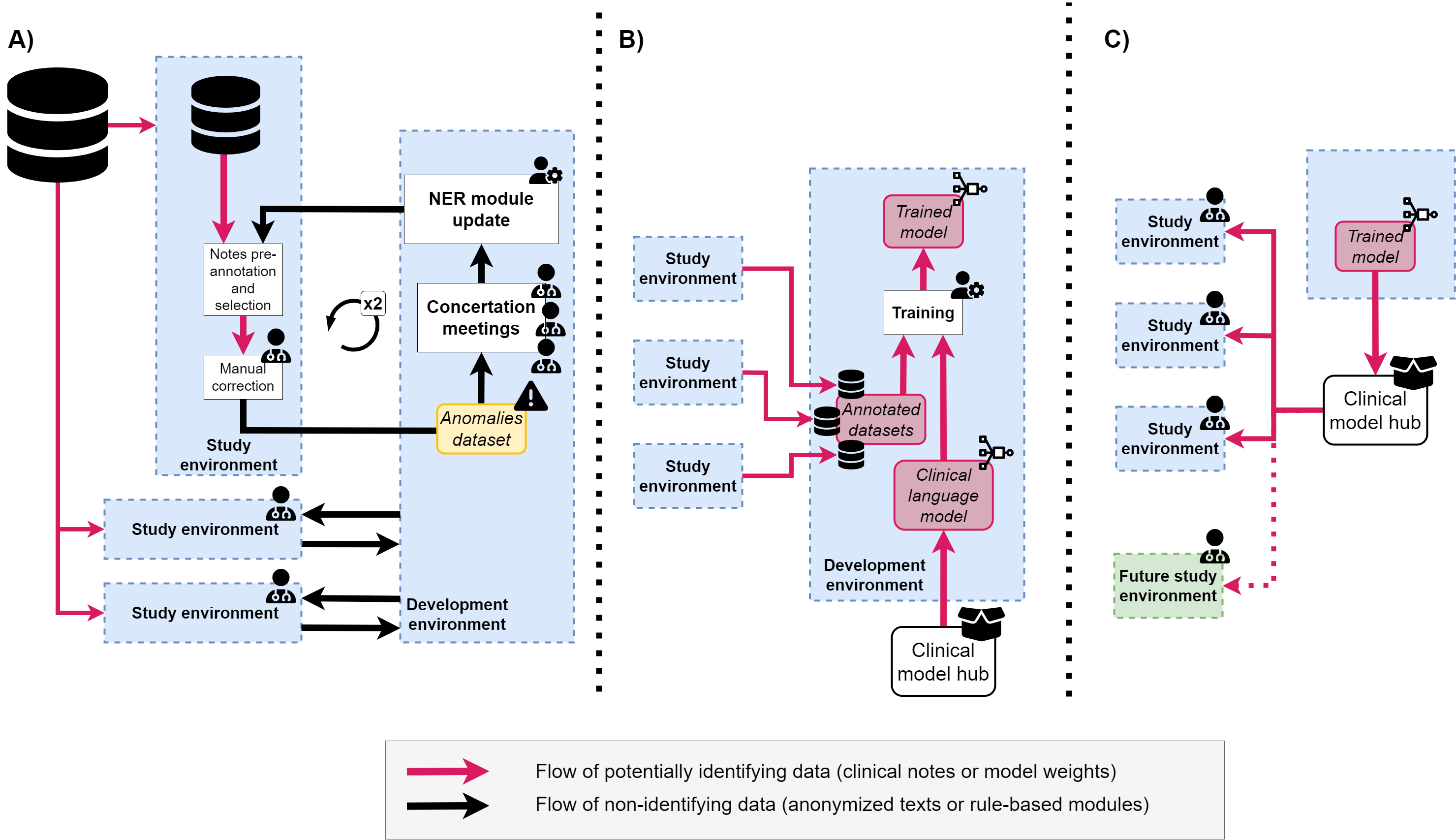

Supplement: ocae069_Supplementary_Data [file ocae069_supplementary_data.zip › ocae069_Supplementary_Data/main_latex_proofred/figures/f2.png]

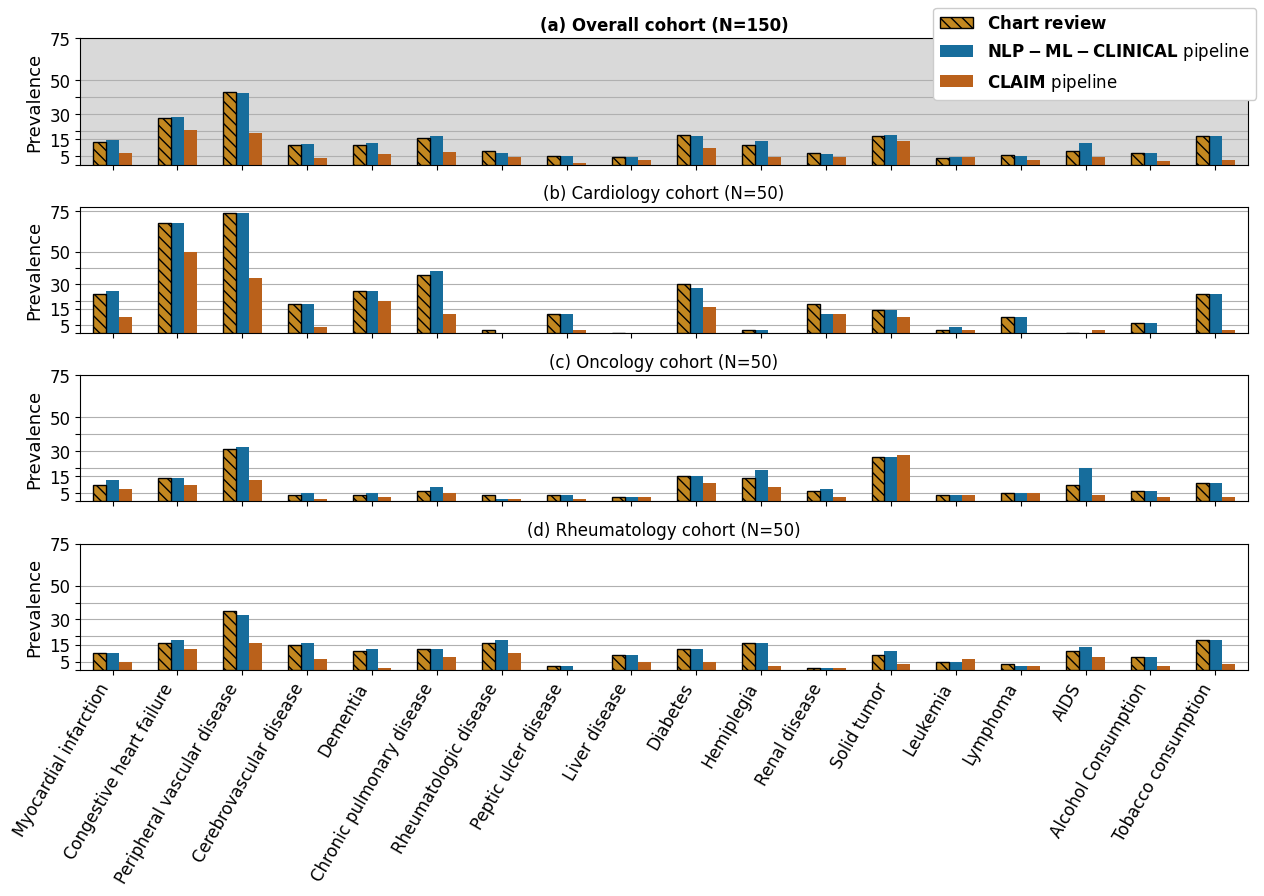

Supplement: ocae069_Supplementary_Data [file ocae069_supplementary_data.zip › ocae069_Supplementary_Data/main_latex_proofred/figures/f3.png]

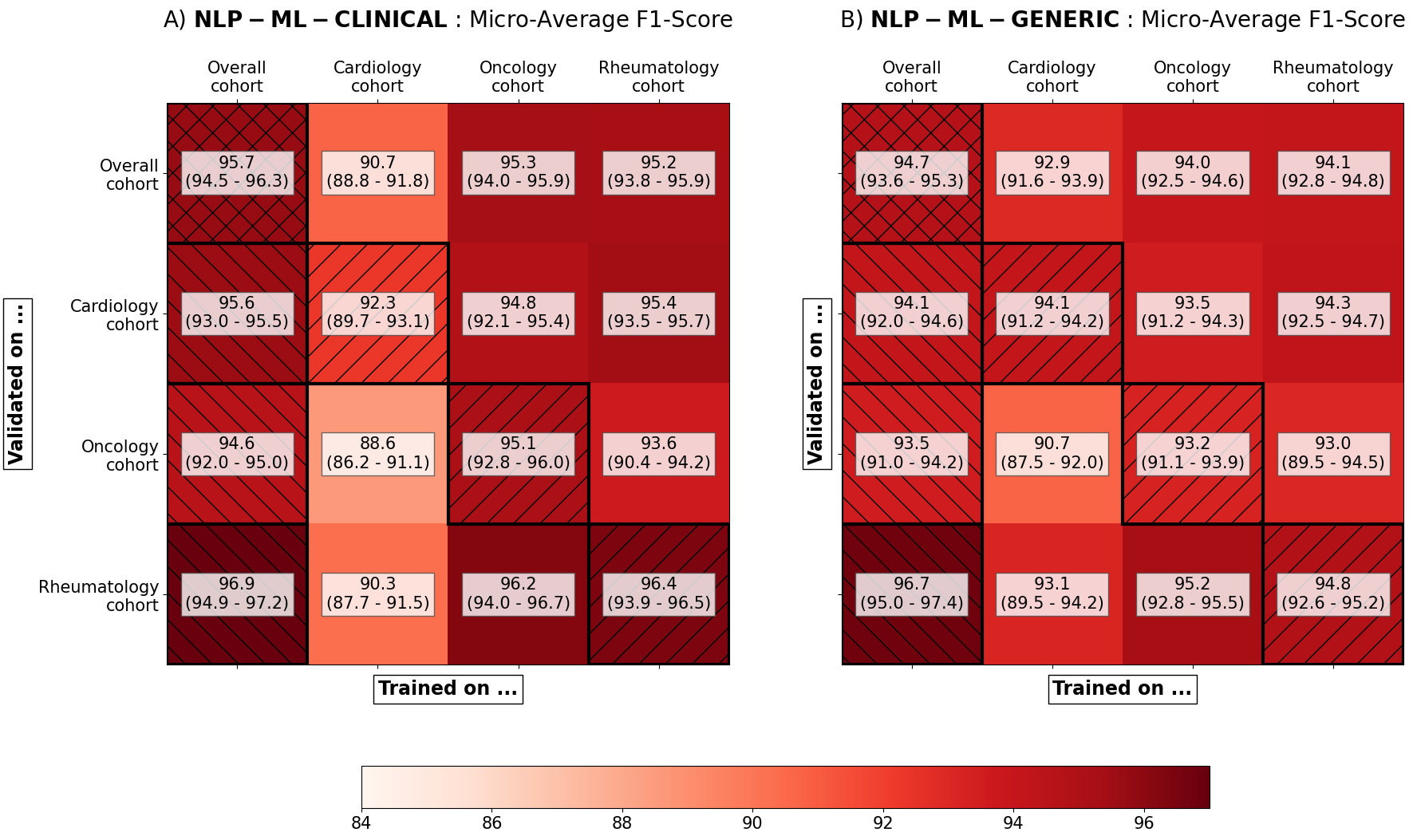

Supplement: ocae069_Supplementary_Data [file ocae069_supplementary_data.zip › ocae069_Supplementary_Data/main_latex_proofred/figures/f4.png]

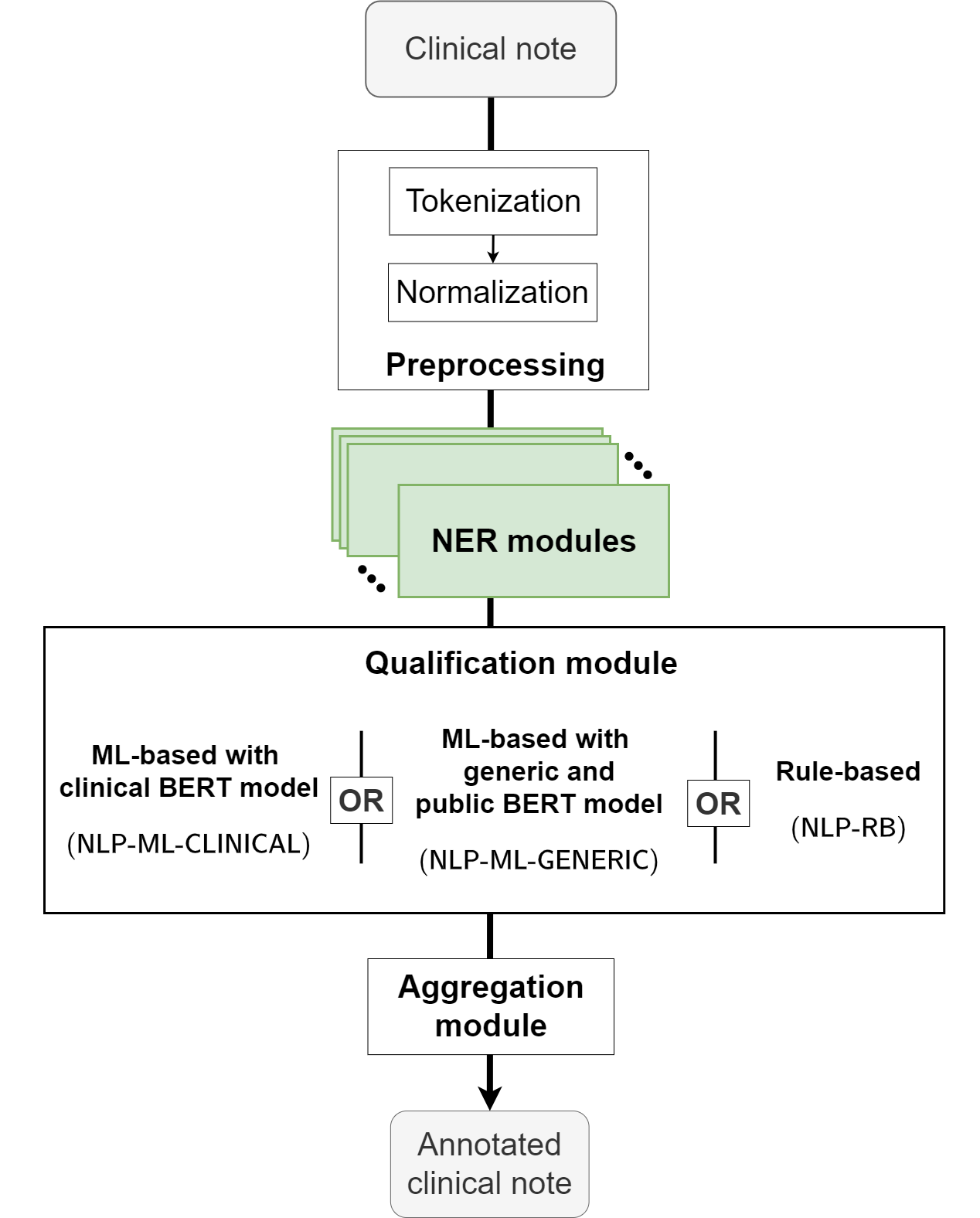

Supplement: ocae069_Supplementary_Data [file ocae069_supplementary_data.zip › ocae069_Supplementary_Data/main_latex_proofred/figures/fchart.png]

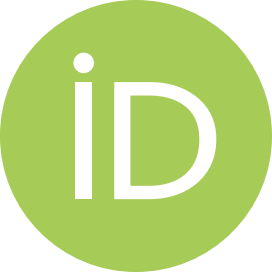

Supplement: ocae069_Supplementary_Data [file ocae069_supplementary_data.zip › ocae069_Supplementary_Data/main_latex_proofred/orcid.pdf]
